# A history of over 40 years of potentially pathogenic free-living amoeba studies in Brazil - a systematic review

**DOI:** 10.1590/0074-02760210373

**Published:** 2022-07-01

**Authors:** Natália Karla Bellini, Otavio Henrique Thiemann, María Reyes-Batlle, Jacob Lorenzo-Morales, Adriana Oliveira Costa

**Affiliations:** 1 Universidade Federal de Minas Gerais, Faculdade de Farmácia, Departamento de Análises Clínicas e Toxicológicas, Belo Horizonte, MG, Brasil; 2 Universidade de São Paulo, Instituto de Física de São Carlos, São Carlos, SP, Brasil; 3 Universidade Federal de São Carlos, Departamento de Genética e Evolução, São Carlos, SP, Brasil; 4 Universidad de La Laguna, Instituto Universitario de Enfermedades Tropicales y Salud Pública de Canarias, Departamento de Obstetricia, Ginecología, Pediatría, Medicina Preventiva y Salud Pública, Toxicología, Medicina Legal y Forense y Parasitología, Red de Investigación Cooperativa en Enfermedades Tropicales, Tenerife, Islas Canarias, Spain; 5 Instituto de Salud Carlos III, Consorcio Centro de Investigación Biomédica en Red MP de Enfermedades Infecciosas, Madrid, Spain

**Keywords:** free-living amoeba, Brazil, literature review, Acanthamoeba, Naegleria, Balamuthia

## Abstract

Free-living amoeba (FLA) group includes the potentially pathogenic genera *Acanthamoeba*, *Naegleria*, *Balamuthia*, *Sappinia*, and *Vermamoeba*, causative agents of human infections (encephalitis, keratitis, and disseminated diseases). In Brazil, the first report on pathogenic FLA was published in the 70s and showed meningoencephalitis caused by *Naegleria* spp. FLA studies are emerging, but no literature review is available to investigate this trend in Brazil critically. Thus, the present work aims to integrate and discuss these data. Scopus, PubMed, and Web of Science were searched, retrieving studies from 1974 to 2020. The screening process resulted in 178 papers, which were clustered into core and auxiliary classes and sorted into five categories: wet-bench studies, dry-bench studies, clinical reports, environmental identifications, and literature reviews. The papers dating from the last ten years account for 75% (134/178) of the total publications, indicating the FLA topic has gained Brazilian interest. Moreover, 81% (144/178) address *Acanthamoeba*-related matter, revealing this genus as the most prevalent in all categories. Brazil’s Southeast, South, and Midwest geographic regions accounted for 96% (171/178) of the publications studied in the present work. To the best of our knowledge, this review is the pioneer in summarising the FLA research history in Brazil.

Free-living amoeba (FLA) is a protozoan group including Excavata and Amoebozoa lineages capable of living free in the environment and alternatively proliferating within a host, therefore named amphizoic amoebas.^1^ *Acanthamoeba* spp, *Naegleria fowleri*, *Sappinia pedata*, *Balamuthia mandrillaris*, and more recently *Vermamoeba vermiformis*, include pathogenic amoebae causative agents of human infections.^1^ ^,^ ^2^ ^,^ ^3^ ^,^ ^4^

FLAs have been ubiquitously isolated around the globe from both natural and artificial environments.^5^ ^,^ ^6^ These organisms share common aspects in their life cycle as the existence of a trophozoite stage able to feed, divide, and move by pseudopodium projection and constrictions of the cytoplasm. Another stage is the cyst, which can withstand adverse conditions as food scarcity, unbalance in pH, salt concentration, and temperature.^6^ *Naegleria* spp. possess an additional flagellate transient form that can escape from harsh environmental conditions.^1^ ^,^ ^7^

FLAs have attracted the interest of human health agencies due to their involvement as opportunistic and non-opportunistic infections that affect the central nervous system (CNS), the cornea, and other organs. CNS infections can be classified either as Primary Amoebic Meningoencephalitis (PAM), an acute infection, caused by *N. fowleri* ^8^ or Granulomatous Amoebic Encephalitis (GAE), a subacute to chronic illness caused by *Acanthamoeba* spp. and *B. mandrillaris*.^2^ ^,^ ^9^ *Acanthamoeba* spp. and *B. mandrillaris* have also been identified in skin lesions and disseminated infections that, similarly to GAE, affect predominantly debilitated or immunocompromised patients.^10^ FLAs can also affect the cornea, causing a progressive, sight-threatening infection termed amoebic or *Acanthamoeba* keratitis (AK), which has *Acanthamoeba* spp as the main etiological agent.^2^ ^,^ ^11^ ^,^ ^12^ *S. pedata* accounts for a single case of encephalitis in humans,^13^ ^,^ ^14^ while *V. vermiformis* has been mainly reported in keratitis cases, most of them in co-infection with *Acanthamoeba*.^4^ Despite the widespread recognition of FLAs as pathogens, infections still result in several deaths or events of visual impairment. These outcomes are mainly associated with the lack of fast and reliable diagnostic methods and effective treatments.^15^ ^,^ ^16^ Besides their importance as human pathogens, FLAs have been investigated in relation to their ecological interaction with the aquatic and soil microbial community. Previous analyses have shown FLA harboring intracellular organisms from the ambient, proposing the amoeba as a vehicle to bacteria, fungi, protozoan, and viruses, the so-called amoeba resistant microorganisms (ARM).^17^ ^,^ ^18^ Through lysing cells, ARMs can disseminate in the environment or a host. Therefore, FLA is referred to as Trojan horses for the microbial community,^17^ ^,^ ^19^ emphasising the significance of investigating its ecological importance throughout the world.^18^

Water-related outbreaks caused by protozoans in Latin America were revised in a survey that pointed out the Brazilian prominence accounting for 30% of the reports (20/66). Comparing the causative agents of these outbreaks, *Acanthamoeba* and *Cyclospora* shared the fourth position in the ranking.^20^ Environmental isolation of FLA in the Brazilian territory relies on recent reports that call attention to the country´s potential on harboring FLAs. Efforts to address gaps in FLA knowledge include developing more reliable diagnosis,^21^ ^,^ ^22^ investigation of factors related to pathogenicity,^23^ ^,^ ^24^ and development of therapeutic approaches.^25^ ^,^ ^26^ Those are just a portion of the literature produced in Brazil on the FLA field, and controversially, there is no literature review devoted to summing up and debating these data critically. Thus, the following central question guided the present literature review: What is the Brazilian research contribution to the free-living amoeba literature until 2020?

*Identification, systematic documentation, and screening of the data* - The data analysis followed the general workflow as suggested by the Preferred Reporting Items for Systematic Reviews and Meta-Analyses (PRISMA) guideline.^27^ Fourteen keywords were defined [Supplementary data (Table II)] and used as queries in the databases Scopus, Web of Science, and PubMed/Medline, all accessed through the CAPES News Portal (www.periodicos.capes.gov.br) between March 22 to 24, 2021. We have defined 13 keywords (#1 to #13) and a geographical region of interest (#14) to select topics of interest [Supplementary data (Table II)].

The search entry varied according to the database, as described in the Supplementary data (Table II), providing an initial dataset composed of 1512 articles (Fig. 1). We managed the literature dataset using Mendeley version 1.17.10^28^ and State of the Art through Systematic Review (StArt) version 3.4 beta.^29^ The StArt tool allowed to identify and subtract duplicate references, corresponding to literature overlaps intra-database and inter-databases. We designed inclusion and exclusion criteria [Supplementary data (Table II)] to accept and reject papers in the first round of data examination. The keywords listed in Supplementary data (Table II) were contrasted against the title, affiliation, and abstract sections of the 475 records selected for screening. We have withdrawn 277 of them (Fig. 1 - Screening step). Next, we examined the main text of the remaining paper according to Supplementary data (Table II - III) criteria. Study limitations included: records available in the gray literature (e.g., google scholar) are absent; the period cutoff excluding the literature made public in 2021; dissertations, letters, and protocols are also absent in this review. Following literature recommendations concerned with diminishing study bias, no language restrictions were applied.^30^ The screening and application of inclusion/exclusion criteria resulted in 178 articles gathering the Brazilian research contribution to the free-living amoeba literature (Fig. 1).

**Fig. 1:**
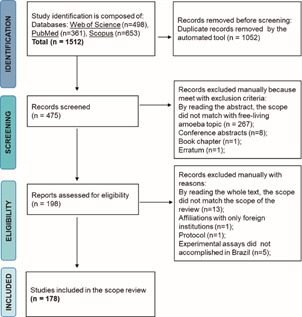
Preferred Reporting Items for Systematic Reviews and Meta-Analyses (PRISMA) flow chart describing the protocol employed in the systematic review and the number of citations (n) retrieved in each step.

Data analysis considered (1) the geographic scope and (2) research domains of the Brazilian literature on FLA topics. A primary analysis exposed a need for two clustering levels concerning the research domain. Based on the central research question addressed in each study we divided the first level into core and auxiliary classes. We settled as *core* the investigations with FLAs as the major topic addressed in all sections of the paper. We sorted some studies including FLAs in any part of the main text, but as a complement to other topics, as *auxiliary*. The second level of clustering consisted of five research domains in which both core/auxiliary papers were categorised: *dry-bench, wet-bench, clinical, environmental and review*.

Publications categorised as wet-bench included *in vitro* or *in vivo* procedures conducted with isolates or cultures without a primary intention of prospecting the environment (environmental) or describing cases (clinical). Dry-bench grouped the bioinformatics-based research whose experimental approach is *in silico*. And review account to literature reviews. We assessed the main text of each paper, and we added the additional two layers of clustering to the data extraction forms. Two researchers have conducted the eligibility checking and data extraction processes to reinforce decisions taken during both steps. We have solved in consensus any uncertainty related to studies inclusion, exclusion, and clustering.

The first and second levels of clustering were displayed in the number or percentage of papers and sorted according to date of publication, federative unit, and FLA species. Occasionally, papers encompassing multiple federative units’ affiliation were scored numerous times and obtained percentages exceeding 100%. However, regardless of the number of authors from the same federative unit, we gave the “1” score.

*FLA research in Brazil had an expansion in the last ten years, and the Southern, South, and Midwest regions lead the contribution* - The FLA topic is an emerging field in Brazil, in which 134 papers from a total of 178 papers (~ 75%) were published in the last ten years of the 1974 to 2020 period (Fig. 2A). The remaining 44 papers (~ 25%) are dated in a time window of 37 years from this period. The data indicate that after 2008, FLA knowledge increased progressively.

Papers classified as the core class accounted for 120 articles predominantly originating from Brazilian scientific institutions in the **S**outheast (SP, MG, ES, RJ), **S**outh (SC, PR, RS), and the Midwest (GO, DF, MS) regions, named here with the acronym **SSM** (Fig. 2B). Although the Southeast region leads the ranking, with ~ 64% of the total reports, the state of Rio Grande do Sul (RS) represents the federative unit with more contributions. The map has also shown studies from the North (PA) and Northeast (PB, and SE) regions with lower frequencies. Overall, it depicts half of the country (13 out of 26 federative unities) contributing on FLA topics sorted as core (Fig. 2B).

**Fig 2:**
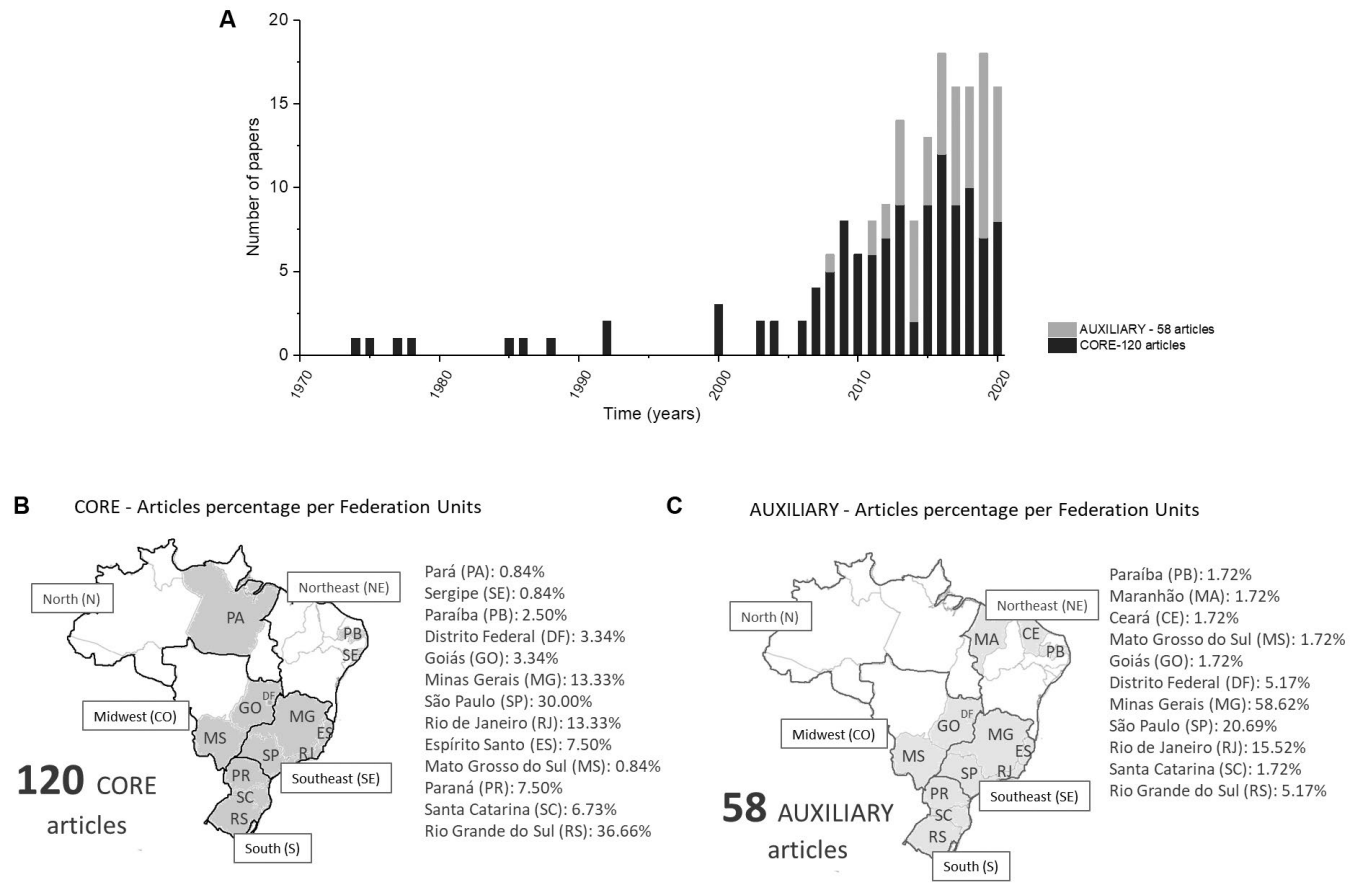
progression and distribution of free-living amoeba (FLA) studies in Brazil. A - Bar graph with the number of core and auxiliary classes of publications per year representing the first level of clustering. Geographical distribution of core (B) and auxiliary (C) classes of papers according to federative units declared in the affiliations. A single paper with multiple federative unities affiliation was scored once per federative unit so that the total percentage resulted higher than 100% in both B and C panels.

A total of 58 articles were classified in the auxiliary class (Fig. 2C). Most of them were originated in universities in the Southeast region, resembling the tendency perceived for the core class of papers (Fig. 2B). Altogether, FLA studies in Brazil have a predominant origin in centres from the SSM region (96% - 171/178). Factors explaining this regional concentration are the heterogeneous funding distributions^31^ and spatial distributions of universities through the country.^32^ Regardless of this trend, the overall landscape of FLA research in Brazil has highlighted half of the federative unities being committed to expanding the knowledge on FLA, therefore reinforcing the country’s relevance as a thriving contributor to the field.

*Wet-bench research predominates in Brazilian FLA literature* - Five research domains composed the second level of clustering in the core and auxiliary classes of papers, as shown in Fig. 3. The research domain classified as wet-bench represents 58 out of 120 core class papers (Fig. 3A), mainly with studies addressing the biochemical characterisation of FLA strains isolated by the authors and commercial lineages (e.g., ATCC strains). Regarding the territorial distribution, wet-bench investigations reproduced the prevalence of the **SSM** region, in which Rio Grande do Sul (RS) and São Paulo (SP) occupied the first and second positions in the ranking with 40.7% and 22% of the published papers, respectively (Fig. 3B).

**Fig. 3:**
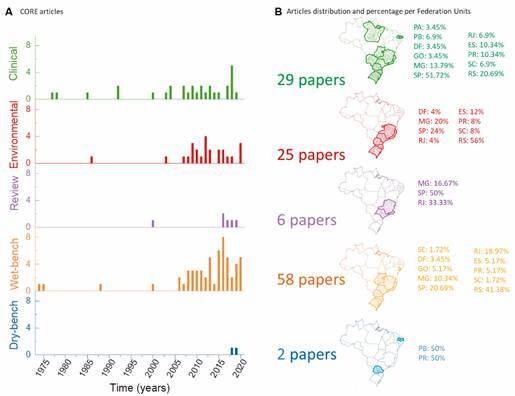
discriminative free-living amoeba (FLA) literature that has FLAs as the central topic (core) in Brazil according to five categories: clinical, environmental, review, dry, and wet-bench. (A) Stacked bar graph comparing the number of papers published per year per category. (B) Territorial scope depicting the percentage of papers per federative unit.

The higher occurrence of clinical assigned reports is on papers describing keratitis (18/29), followed by encephalitis (7/29). Fewer reporting cases of disseminated diseases (1/29), skin infections (1/29), besides the presence of FLA in urine and feces (2/29), were reported. Summarising the Brazilian publications on FLA distribution, the environmental category identified 25 out of 120 papers in the core class.

The remaining 58 papers sorted in the auxiliary class included 60% (n = 34) of wet-bench research [Supplementary data (Figure)], reinforcing the predominance of this research domain in FLA national studies. Review, clinical and environmental tags account for 13, 6, and 5 papers, respectively, out of the 58 in this class, while no study was classified as dry-bench. Once again, the Southeast region leads the number of studies, covering topics ranging from waterborne protozoa to more specific issues as virus replication.

*Acanthamoeba leads the ranking of FLA studies in Brazil* - The *Acanthamoeba* genus was predominant in 81% (144/178) of papers [Supplementary data (Table I)], a tendency depicted in clinical, environmental, wet-bench and review categories of both the core and auxiliary classes (Fig. 4). For the dry-bench category, both *Acanthamoeba* and *Naegleria* shared the same number of publications (one paper each) in the core class.

**Fig. 4:**
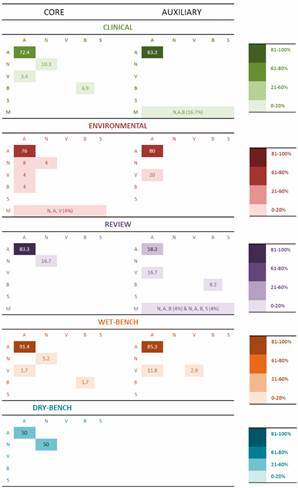
the diversity of the free-living amoeba (FLA) genus per category and class of references. From intense to pale shades, the color spectrum, indicates the prevalence of FLA genera per number of papers. A, N, V, B, and S are *Acanthamoeba*, *Naegleria*, *Vermamoeba*, *Balamuthia*, and *Sappinia*, respectively. M: multiple topics (two or more FLA genera included in the main scope).

To a lesser extent, some papers addressed two FLA genera in a single research, as revealed by combinations of *Vermamoeba* and *Acanthamoeba* in the clinical category, as well as *Naegleria* and *Acanthamoeba* in the environmental category, both within the core class (Fig. 4). Multiple emphases occurred in papers discussing more than two FLA genera in a single paper, for example, *Naegleria*, *Acanthamoeba,* and *Balamuthia* identified in clinical publications of the auxiliary class (Fig. 4). Regardless of the single or multiple FLA genus in the same article, those classified in the environmental category accounted for *Acanthamoeba*, *Vermamoeba*, *Balamuthia*, and *Naegleria* reports in the Brazilian territory (Fig. 4). *Sappinia* was mentioned only in two papers classified as reviews in the auxiliary class.^33^ ^,^ ^34^ The predominance of *Acanthamoeba*-related investigations is an expected finding considering that this species is the most frequent FLA in the environment^2^ and presents higher viability than *Naegleria* in dust samples.^35^ In addition, while other pathogenic species of FLAs are involved in rare brain infections, *Acanthamoeba* can cause both cerebral and corneal AK infections, the latter showing an increasing number of cases in recent years.^2^

*Diversity of approaches in wet-bench references* - The wet-bench category included approaches that varied from morphological characterisation to the development of diagnostic and therapeutic tools. Biochemical assays aiming to isolate and characterise nuclear and mitochondrial DNA of *Acanthamoeba* were the pioneer reports on FLA in Brazil, being published in 1974 and 1975.^36^ ^,^ ^37^ A long gap of reports on wet-bench research occurred until 1992 when a study reported an isoenzymatic and antigenic assessment towards classifying *Acanthamoeba* isolates in groups I, II and III.^38^ Further, the randomly amplified polymorphic DNA (RAPD) technique was indicated as a suitable strategy to rapid genotyping new isolates.^39^ More recent studies adopted high throughput sequencing with bioinformatics pipelines to decipher nuclear and mitochondrial DNA of *Acanthamoeba* and *Vermamoeba* strains.^40^ Proteomics and metabolomics analysis are also represented by studies devoted to characterising *Acanthamoeba*, which provided informative molecular elements with the potential to be investigated as drug targets or as epitopes for antibodies in diagnostic tools.^41^ ^,^ ^42^ ^,^ ^43^ Additional targets for detection purposes are cation:proton antiporter protein family (CPA) and a calreticulin extracellular domain.^44^ ^,^ ^45^ The former was identified through the monoclonal antibody mAb3, which positively reacted with several *Acanthamoeba* pathogenic strains.^44^ The latter study described the use of a recombinant polypeptide, produced from a predicted sequence of *A. castellanii* calreticulin, which was recognised by sera from rats with GAE in the ELISA test.^44^ These contributions meet the urgent demand for faster detection techniques since late diagnosis is a factor contributing for the high mortality or visual sequelae of FLA infections.^1^ ^,^ ^2^ ^,^ ^6^ ^,^ ^8^Other *Acanthamoeba* wet-bench research is concerned with culture manipulation, as the proposal of strategies for prolonging culture storage^46^ and removing contaminants from cultures.^47^ Additional approaches included investigating the role of phosphate transporters in metabolism,^48^ description of ultrastructural cyst wall morphology,^49^ and genomic features.^50^ ^,^ ^51^ Once the primary status of FLAs is not parasitic, a relevant issue in the research field is understanding factors associated with the pathogenic behavior. Brazilian studies explored *in vitro Acanthamoeba* properties, such as the interaction of trophozoites with the host cell or the extracellular matrix.^24^ ^,^ ^52^ ^,^ ^53^ ^,^ ^54^ Contact-independent elements were also included, with contributions that described released proteases through the zymographic technique, indicating the serine protease class as the predominant type produced by *Acanthamoeba*.^55^ ^,^ ^56^ ^,^ ^57^ ^,^ ^58^ Of note, an additional contribution showed for the first time that *Acanthamoeba* produces and releases extracellular vesicles (EVs) that carry proteases and can be/are taken up by target cells, causing cytotoxicity.^59^ Apart from the entirely *in vitro* investigations aforementioned, a study adopted a rat model to induce systemic and cerebral acanthamoebiasis as a strategy to reactivate the virulence of *Acanthamoeba* cultures.^60^

Efforts to develop novel and improve existing therapies to combat FLA infections have been performed in Brazil, including tests with both first-line options and novel compounds. Polyhexamethylene biguanide and chlorhexidine digluconate were evaluated alone or combined against *Acanthamoeba* cysts.^61^ Those biguanides and diamidines (e.g., propamidine and hexamidine isethionate), are currently the main therapeutic lines for *Acanthamoeba* keratitis.^62^ As they exert cytotoxicity to the cornea, searching for new amoebicidal products is still necessary. Thirteen studies proposed in Brazil included dose-response assays with natural and synthetic substances in this direction. Examples are bacteriocin-like compounds,^63^ alpha-helical and beta-hairpin antimicrobial peptides,^64^ S-nitrosothiols,^65^ silver nanoparticles,^66^ plant extract, or essential oils directly assayed.^67^ ^-^ ^73^ Two studies have explored alternatives such as photodynamic therapy by using riboflavin and curcuminoids as photosensitisers for treating *Acanthamoeba* keratitis.^74^ ^,^ ^75^ The effectiveness of contact lens multipurpose solutions against *Acanthamoeba*, a topic debated by the FLA community,^76^ has been also investigated in Brazil,^77^ ^,^ ^78^ including the suggestion of imidazolium salt, an anti-amoebic substance, as a suitable option for lens storage.^79^ Alternatively, therapeutic strategies were concerned with molecules essential to cell division, energy production, cell differentiation, or movement. Examples consist of two Brazilian studies testing the role of small interfering RNA (siRNA) carried in liposomes as an anti-amoebic agent by *in vitro* and *in vivo* assays.^26^ ^,^ ^80^ Even though mentions of other FLA species were less frequent than *Acanthamoeba*, *B. mandrillaris* is represented in a study describing the amoeba-host cell interaction.^81^ *Naegleria* was the focus of three reports; in one of them, mass spectrometry (MALDI-TOF MS) was proposed as a tool to discriminate *N. fowleri* from non-pathogenic species (e.g., *N. italica*, *N. jadini*, *N. gruberi*).^82^ In two papers, proteins of *Naegleria* were characterised by biophysical approaches, providing insights about its metabolism.^83^ ^,^ ^84^

Wet-bench Brazilian research also included ecological information on FLA and amoebic resistant microorganisms (ARMs). Some reports discuss interactions of FLAs with fungi and bacteria, addressing both endosymbionts and pathogens of concern in human health.^19^ ^,^ ^85^ ^-^ ^90^ Another type of ARMs studied in Brazil comprises human adenovirus^91^ and giant viruses such as the Tupanvirus, isolated from *V. vermiformis* and *A. polyphaga* mimivirus.^92^ ^,^ ^93^ We discussed below other reports on giant virus classified in the auxiliary class. Apart from intra-amoebic microorganisms, a wet-bench study investigated the ecological relation between amoeba and mosquitoes, demonstrating for the first time that *A. polyphaga* can infect *Aedes aegypti*.^94^ Moreover, the role of FLA as vehicles of pathogenic microorganisms debated worldwide^95^ ^,^ ^96^ ^,^ ^97^ and also present in Brazilian surveys^85^ ^,^ ^88^ ^,^ ^91^ ^,^ ^98^ reinforce the need of monitoring FLA presence in the environment.

*Clinical references and the risks of FLA infections for human health in Brazil* - As shown in Fig. 3, 29 out of 178 references were classified in the clinical category, including mainly *Acanthamoeba* keratitis,^99^ ^-^ ^104^ PAM,^105^ and GAE,^106^ reports (Table I).

**TABLE I:** List of cases of free-living amoeba (FLA) infections in Brazil - from 1977 to 2020

#	Year	Case / Patient - Outcome^*a*^	Identified FLA	Method^*b*^	Ref
1	1977	Encephalitis / Human - Death	*Acanthamoeba*; *Hartmannella*	M	^106^
2	1978	Encephalitis / Human - Survived	*Naegleria* spp.	M	^114^
3	1992	Encephalitis / Human - Death	*Leptomyxid amoeba*	M, I	^152^
4	2007	Encephalitis / Human - Death	*Balamuthia mandrillaris*	M, I	^154^
5	2010	Encephalitis / Human - Death	*Balamuthia mandrillaris*	M, I, D	^116^
6	2012	Encephalitis / Cattle - Death	*N. fowleri*	M, I	^105^
7	2019	Encephalitis / Cow - Death	*N. fowleri*	M, I	^107^
8	2000	Keratitis / Human	*A. astronyxis*	M, D	^39^
9	2003	Keratitis / Human	*Acanthamoeba* spp*.*	M	^99^
10	2004	Keratitis / Human	*Acanthamoeba* spp*.*	M	^100^
11	2004	Keratitis / Human	*Acanthamoeba* spp*.*	M	^153^
12	2007	Keratitis / Human	*Acanthamoeba* spp.	M	^22^
13	2008	Keratitis / Human	*Acanthamoeba* spp.	M	^101^
14	2009	Keratitis / Human	*Acanthamoeba* spp.	M	^102^
15	2011	Keratitis / Human	*A. castellanii*	M	^155^
16	2011	Keratitis / Human	*Acanthamoeba* T4	M, D	^74^
17	2013	Keratitis / Human	*Acanthamoeba* T4, T11, T2 and T4/T2; T4/T13; T4/T16	M, D	^103^
18	2013	Keratitis / Human	*A. castellanii*, *A. rhysodes*	M, I, D	^61^
19	2017	Keratitis / Human	*A. castellanii* T4	M, D	^104^
20	2017	Keratitis / Human	*Acanthamoeba* spp.	M, D	^21^
21	2018	Keratitis / Human	*Acanthamoeba* spp.	M	^87^
22	2018	Keratitis / Human	*Acanthamoeba* spp.	M	^115^
23	2018	Keratitis / Human	*Acanthamoeba* T4	M, D	^108^
24	2018	Keratitis / Human	*Acanthamoeba* spp T4 (co-infection)^*c*^	M, D	^109^
25	2018	Keratitis / Human	*Acanthamoeba* T4	M, D	^110^
26	2014	Skin infection / dogs - Survived	*Acanthamoeba* T3, T4, T5, T16	M, D	^111^
27	2015	Disseminated amoebic disease / dogs - Death	*Acanthamoeba* spp.	M, I	^112^
28	1985	Other / human feces	*Acanthamoeba* spp., *Hartmannella* spp.	M	^151^
29	2009	Other / urine samples	*Acanthamoeba* spp.	M	^113^

*a*: the disease outcome (survival/death) was not applicable for both keratitis and other clinical samples; *b*: M - microscopy (morphology - M); immunohistochemistry (I); DNA based approach (D); *c*: co-infection case (*Acanthamoeba* spp and *Candida albicans*). Ref: references.

FLA infections reported in Brazil present the occurrence of *Naegleria* spp. in encephalitis cases,^105^ ^,^ ^107^ and *Acanthamoeba* spp. as the commonest genus of keratitis,^108^ ^-^ ^111^ skin infections, disseminated disease, and even encountered in human feces^112^ and urine.^113^ *Vermamoeba* and *Balamuthia* were reported in two records, and no *Sappinia* cases were described (Table I). Of seven encephalitis cases, only one describes the recovery of the patient.^114^ It is dated from 1978, reporting the recovery of a 14-year-old boy with a habit of bathing in lagoons. Water samples were collected, confirming the presence of *N. fowleri*.^114^

*Acanthamoeba* infections, basically keratitis, represent the highest incidence of amoeba contaminations in Brazil. In this group, investigations dated after 2010 described molecular approaches (Table I-D), indicating this technology became more accessible in the laboratory routine.

The widely debated misdiagnosis of FLA infection^62^ has also been reported in the Brazilian literature, as discussed in a retrospective study of cases of amoebic keratitis, in which the herpes virus was the most frequent initial suspicion.^115^ The same occurred for amoeba brain infections, in which a fatal case in Brazil was first hypothesised and treated for viral meningitis.^116^ Despite that, the overall tendency to rapidly and accurately diagnose FLA proposed in Brazilian surveys may decrease the misdiagnosis. On the other hand, monitoring of intra-FLA resistant microorganisms is an issue of concern worldwide^1^ ^,^ ^97^ ^,^ ^117^ that is still absent in the clinical category of Brazilian papers.

*Environmental reports and the assessment of FLA as a water quality marker in Brazil* - *Acanthamoeba*, *Naegleria*, *Vermamoeba*, and *Balamuthia* were detected in Brazilian habitats, including university buildings, hospitals, swimming pools, tap and freshwater, dust, soil samples, and inside insects^118^ ^-^ ^131^ (Table II).

**TABLE II:** A summary of the environmental isolations of free-living amoeba (FLA) in Brazil, from 1986 to 2020

Year	Sample	FLA identification	Method^*a*^	Ref
1986	Lake	*N. fowleri*	M,I	^132^
2003	Hospitals	*Acanthamoeba*, *Naegleria*	M	^118^
2007	Public hospitals	*Acanthamoeba* spp.	M	^35^
2008	Contact lenses cases	*Acanthamoeba* spp.	M, D	^119^
2009	University buildings	*Acanthamoeba*; *Naegleria*	M, I	^133^
2009	Water samples	*Acanthamoeba* ACC01	M,D	^135^
2009	Swimming pools	*Acanthamoeba* spp.	M,D	^120^
2010	Hospital localities	*Acanthamoeba* T4, T5, T3	M	^121^
2010	Public hospitals	*Acanthamoeba* spp.	M,D	^126^
2011	Tap water	*Acanthamoeba* T2,T6, T4	M,D	^127^
2012	Soil and water samples	*Acanthamoeba* T4, T5, T2-T6	M	^128^
2012	*Aedes aegypti* larvae	*Acanthamoeba* T4, T3, T5	M,D	^129^
2012	Soil and water samples	*Acanthamoeba* T7, T8, T9, T17	M,D	^130^
2012	Public hospitals	*A castellanii*, *A. polyphaga*, *A. lenticulata*	M,D	^131^
2013	Bromeliads leaves	*Acanthamoeba* T4, T2/T6, T16	M,D	^120^
2013	Dust of domicile	*Acanthamoeba* spp.	M,I	^121^
2015	Air conditioned samples and contact lenses cases	*Acanthamoeba* T4, T5, T3	M,D	^98^
2015	Mineral bottled water	*Acanthamoeba* T5, T4, T11	M,D	^122^
2016	Hot tubes, swimming pools	*Acanthamoeba* T3, T5, T4, T15	M,D	^123^
2016	Swimming pools	*Acanthamoeba* spp.	M,D	^91^
2017	Air conditioned cooling tower samples	*Acanthamoeba* spp T4, T5, *Vermamoeba vermiformis*, *Naegleria* sp., *N. australiensis*	M,D	^136^
2018	Dust, sewage, soil, biofilm, sea samples	*Acanthamoeba* T1, T15 and T18	M,D	^134^
2020	Air conditioned in hospitals	*Acanthamoeba* T3, T4, *Balamuthia mandrillaris*	M,D	^138^
2020	River water	*N. philippinensis*, *N. canariensisi*, *N. australiensis*, *N. gruberi*, *N. dobsoni Hartmannella* spp.	M,D	^137^
2020	Air conditioned in hospitals	*Acanthamoeba* T4, T5, T11.	M,D	^157^

*a*: M - microscopy (morphology - M); immunohistochemistry (I); DNA based approach (D). Ref: references.

The first isolation in the environment is dated from 1986 and pointed out the presence of *N. fowleri* in an artificial lake in Rio de Janeiro city, state of Rio de Janeiro, the Southeast region.^132^ Dust samples from hospitals and university buildings yielded positive results for *Naegleria* spp, in the municipalities of Presidente Prudente and Santos, respectively, both in São Paulo state, the Southeast region.^118^ ^,^ ^133^ Although based on morphological analysis, a study indicated the *N. fowleri* presence by its ability for supporting 43ºC and flagellating.^1^ ^,^ ^133^

*Naegleria* and *Vermamoeba (Hartmanella)* identifications were less frequent compared to *Acanthamoeba*,^134^ ^,^ ^135^ but they were found coexisting in the same habitats (Table II).^136^ ^,^ ^137^ Environmental findings of *B. mandrillaris* were reported to be associated with *Acanthamoeba* populations in air conditioning systems of hospitals.^138^ Across the globe, FLAs have been sampled in natural habitats related to water and soil^139^ ^,^ ^140^ ^,^ ^141^ besides extreme environments such as treatment water plants, chlorinated swimming pools, hot spring water, and ice samples.^142^ ^,^ ^143^ ^,^ ^144^ Global warming can favor the spreading of thermophilic pathogenic FLA^145^ ^,^ ^146^ and intra-FLA pathogenic bacteria.^147^ In Brazil, a tropical zone directly exposed to global warming effects,^148^ the presence of potentially pathogenic FLA strains described in water-related samples (Table II) can pose a health concern. Although the country’s regulatory legislation of water quality does not address FLA risks for humans,^149^ the need for research on tools to detect them is identified in the global literature.^150^

*Progression of* FLA *detection and characterisation tools in Brazil* - With the expansion of studies on FLA in the country, methods for detection and characterisation of environmental and clinical samples have improved, with a tendency to adopt molecular approaches over time (Fig. 5).

**Fig. 5:**
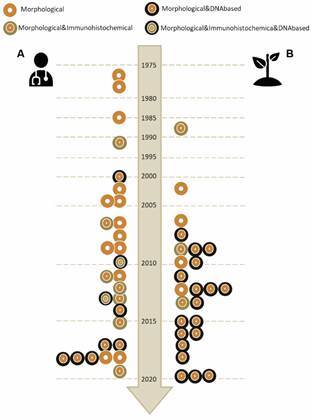
progression of the methods used in Brazil to monitor FLA presence in clinical (A) and environmental (B) samples.

Microscopy-based methods for detection and characterisation were adopted in all studies (54/54) and in some, (17/54) it was the only option to identify the FLA. This approach predominates until 2005 (Fig. 5). Between 2010 and 2020, 23 out of 34 FLA environmental and clinical investigations included molecular-based tools for FLA detection, in contrast with five “morphological” and six “Morphological & Immunohistochemical” based analyses. Moreover, it is worth noting that DNA-based investigations were more prevalent on environmental (18/25) than clinical (9/29) references (Fig. 5).

Overall, the studies included microscopy-based investigations combined with hematoxylin-eosin (HE),^151^ ^,^ ^152^ calcofluor and Giemsa stains.^153^ Anti-*B. mandrillaris* antisera has been used for immunofluorescence purposes.^116^ ^,^ ^154^ Before performing microscope slides, environmental and clinical samples were often cultured in non-nutritive agar plates^155^ with an additional supply of bacteria such as *Escherichia coli*,^153^ *Enterobacter*,^22^ ^,^ ^153^ *Aerobacter aerogenes*,^151^ *Micrococcus luteus* and *Pseudomonas aeruginosa*.^156^ Osmotolerance and thermotolerance assays have been performed in NNA plates for checking pathogenicity.^124^ ^,^ ^128^ ^,^ ^157^ Molecular-based approaches comprised PCR assays in which 18S rDNA regions^50^ ^,^ ^158^ or ITS regions^159^ were used to genotyping FLA isolates, and the V3 region of 16S rDNA of bacteria was used to detect intra-FLA bacteria.^98^ Among the wet-bench category of papers, one study has employed a high-throughput sequencing (HTS) methodology to analyse mitochondrial gene sequences elucidative to investigate diversity in Amoebozoa.^40^

*Reviews and dry-bench studies in the core class* - A group of literature reviews covered a diversity of FLA-related topics. The first literature review dates from 2000, and it is devoted to reporting predisposing factors commonly associated with amoebic keratitis, including clinical aspects, diagnosis strategies, and therapeutic options.^160^ Two comprehensive reviews addressed mechanisms developed by intra *Acanthamoeba* microorganisms to enhance their survival and multiplication chances.^161^ ^,^ ^162^ Since several ARMs are capable of proliferating in FLAs and in the human host, other reviews discussed the hypothesis that ARMs can use the amoeba cell to gain virulence before inhabiting humans.^161^ Subsequent work discussed clinical aspects of *N. fowleri* infection and debated therapeutic challenges when treating PAM patients.^159^ The latest literature review was published in 2019, describing the role of extracellular vesicles of bacteria, fungi, and protozoans with a particular emphasis on *A. castellanii*.^163^

Finally, the dry-bench references gathered relevant information for ARM monitoring. For example, a study accessed genomic signatures shared by *Acanthamoeba* entoorganisms by using oligonucleotide relative frequencies (OnRF) and relative codon usage (RCU) approaches.^164^ An additional publication described the tridimensional structure of glyceraldehyde-3-phosphate dehydrogenase (GAPDH) of *N. gruberi*.^165^

*Auxiliary category* - The auxiliary class grouped 58 references on broad subjects that also embraced FLA topics. It includes virus replication,^166^ ^,^ ^167^ ^,^ ^168^ ^,^ ^169^ microbial keratitis,^170^ ^,^ ^171^ main aspects of central nervous disorders^172^ ^,^ ^173^ ^,^ ^174^ and cutaneous infections,^175^ reaction of microorganisms against phototherapies,^176^ among others.

Prospection surveys concerned with waterborne protozoans have shown *Giardia* and *Acanthamoeba* in saline water and oysters samples, highlighting both as a concern for human health.^177^ Similarly, a foodborne parasite-based examination in salad samples of Brazilian restaurants showed *Acanthamoeba* presence ranking in the first position with 23.5% identifications.^178^ Although this identification suggests the importance of food as a source of free-living amoeba, as far as we know, there is no evidence of infections elicited by ingesting FLA.

A study on keratitis has indicated the following groups as etiological agents of corneal infections: bacteria (*Staphylococcus*, *Streptococcus*, *Enterobacter*), fungi (*Candida*, *Rhodatorula*), and protozoan groups (*Acanthamoeba*) as possible etiological agents of corneal infections.^179^ From 2007 to 2014, another study examined 242 patients from a specialised eye hospital whose results indicated bacteria as the first cause of keratitis infection, and the successive positions in the ranking were occupied by fungi and *Acanthamoeba* infections.^180^

Concerned with treatments, the auxiliary literature has investigated UV light and natural compounds acting as photosensitisers for treat a diversity of parasitic and microbial infections, including *Acanthamoeba* keratitis.^181^ A related survey concerned with the ranges of visual acuity recovery has examined the efficacy of Boston keratoprothesis type I for *Acanthamoeba* keratitis.^182^ Other contributions are assays to investigate the effectivity of a biosurfactant on *Candida* and *Acanthamoeba* ^183^ and anti-amoebic action of metabolites from *Penicillium*.^184^

Sixty-seven percent (39/58) of the auxiliary references describe investigations specific to ARMs. Fungi and FLA interactions addressed *Trichophyton rubrum*,^90^ *Fusarium*,^85^ *Sporothrix*,^185^ *Paracoccidioides* spp.,^186^ and *Cryptococcus* species,^183^ ^,^ ^187^ ^,^ ^188^ all associated with *Acanthamoeba*. Some of these reports highlight that fungi-FLA interactions in the environment mimic those found between fungi and host cells, possibly contributing to the development of virulence and immune escape strategies.^187^ ^,^ ^188^ ^,^ ^189^

One study identified *Pseudomonas* in *Acanthamoeba* isolates.^86^ Another survey adopted a co-culture model between this FLA genus and methicillin-resistant *Staphylococcus aureus*, showing mutual effects on cell growth or differentiation.^190^ A third report investigated the role of a K(+) transporter gene in the nutrient uptake and replication of *Legionella pneumophila* inside *A. castellanii*.^191^

Several reports on ARMs (30/39) have comprised the giant viruses, placing this topic as a highlight in the auxiliary category. Relevant contributions included new virus species and lineages described after prospection in Brazilian environments.^192^ ^-^ ^199^ Exploring variable aspects of interaction, a set of reports addressed the morphological description of viral attachment, replication, and release^200^ ^-^ ^207^ to the analysis of gene expression regulating the host cell cycle.^92^ ^,^ ^93^ ^,^ ^204^ ^,^ ^208^ Reviews and editorials^209^ ^,^ ^210^ ^,^ ^211^ ^,^ ^212^ described methods for the isolation, culture^213^ ^,^ ^214^ ^,^ ^215^ and antiviral biocides assays^216^ ^,^ ^217^ also compose the auxiliary list of papers. Additional studies debated virus lateral gene transfer,^212^ ^,^ ^218^ or in co-infections with virophages^219^ that may likely contribute to genetic variation to the amoeba host, and even the opposite situation, in which viruses are capable of integrating amoeba genes into its genome.^220^ ^,^ ^221^ Finally, one dry-bench study in the auxiliary class comprises a review that explored the sexual process in Eukaryotes, discussing the expression of meiosis genes in *Acanthamoeba*.^222^ Altogether, the auxiliary class references added valuable information to the knowledge about FLA and its relationship with other organisms.

So far, no literature review was devoted to critically analysing the Brazilian publications on the FLA topic, although two^18^ ^,^ ^223^ out of four literature reviews on *Vermamoeba* spp,^224^ *Naegleria*,^15^ ^,^ ^223^ and *Acanthamoeba* ^18^ mentioned the country, several Brazilian reports are missing in the latest literature reviews.

*Conclusions and directions* - The present work is the first literature review devoted to critically integrating and debating FLA data produced in Brazil. The data described here outlined the main scientific issues related to FLA research in Brazil since the first publication in 1974 and covering the last 46 years. Even though several FLA genera were reported during this study period, most papers were related to *Acanthamoeba*, and the wet-bench investigations predominated.

Regarding the clinical reports, we believe there are many under-reported cases of FLA infections in Brazil, including keratitis and encephalitis cases, as previously suggested by retrospective studies. In addition, the possibility of pathogenic intra-amoebic microorganisms isolated from clinical FLA strains increases the potential of FLA-ARM interaction to be harmful to human health. Due to that, it is urgent to narrow the relations between researchers and clinicians, universities, hospitals, and medical laboratories in the country, aiming to close monitoring cases of FLA diseases, or even bacterial, fungal, and viruses infections likely carried through amoebas.

The Brazilian epidemiologic surveys on FLA in water collections did not consider the limnologic data on the samples. It is advisable for policymakers and environmental regulatory departments to integrate the efforts towards improving the knowledge on amoeba distribution. In this direction, understanding the correlation between amoeba presence and environmental characteristics may contribute to better evaluating public health risk areas. Finally, national funding opportunities and distribution across Brazil are extremely important to boost the spread of FLA research in the country and contribute to public health awareness.
