# Supplementary material for: A history of over 40 years of potentially pathogenic free-living amoeba studies in Brazil - a systematic review
Source: Mem Inst Oswaldo Cruz. 2022 Jul 1;117:e210373. doi: 10.1590/0074-02760210373 (PMC9252135; doi:10.1590/0074-02760210373)
Supplement: Supplementary file 1 [file 1678-8060-mioc-117-e210373-s.pdf]

TABLE I

List of references included in the present literature review

Year	Title	Category[a]	Target FLA[b]	#[c]
**CORE**				
1974	Isolation of nuclei and characterization of nuclear DNA of *Acanthamoeba castellanii*	W	A	(1)
1975	Characterization of the genome of the small free-living amoeba *Acanthamoeba castellanii*	W	A	(2)
1977	Primary amoebic meningoencephalitis: first case from Latin-America [Meningencefalite por ameba de vida livre apresentacao do primeiro caso latino-americano]	C	N	(3)
1978	Primary amebic meningoencephalitis - case-report	C	N	(4)
1985	Free-living amoebae in human intestine: evidence of parasitism [Amebas de vida livre no intestino humano evidencias de parasitismo]	C	A, W	(5)
1986	Isolation of *Naegleria fowleri* from a lake in the city of Rio de Janeiro, Brazil	E	N	(6)
1988	*Acanthamoeba culbertsoni*: susceptibility to soft contact lens disinfection systems	W	A	(7)
1992	*Acanthamoeba healyi* N. sp. and the Isoenzyme and Immunoblot Profiles of *Acanthamoeba* spp., Groups 1 and 3	W	A	(8)
1992	Granulomatous amoebic encephalitis due to leptomyxid amoebae: Report of the first Brazilian case	C	A	(9)
2000	*Acanthamoeba keratitis* [Ceratite por *Acanthamoeba*]	R	A	(10)
2000	Proteinase activities in total extracts and in medium conditioned by *Acanthamoeba polyphaga* trophozoites	W	A	(11)
2000	Random amplified polymorphic DNA profiles as a tool for the characterization of Brazilian keratitis isolates of the genus *Acanthamoeba*	C	A	(12)
2003	Diagnosis of *Acanthamoeba* keratitis with confocal microscopy [Microscopia confocal no diagnóstico de ceratite por *Acanthamoeba*]	C	A	(13)
2003	Isolation of potencially pathogenic free-living amoebas in hospital dust	E	A,N	(14)
2004	Confocal microscopy in early diagnosis of *Acanthamoeba* keratitis	C	A	(15)
2004	Conjunctivitis presumably due to *Acanthamoeba* [Conjuntivite presumível por *Acanthamoeba*]	C	A	(16)
2006	The interaction between *Acanthamoeba polyphaga* and human osteoblastic cells *in vitro*	W	A	(17)
2006	The interaction between the amoeba *Balamuthia mandrillaris* and extracellular matrix glycoproteins *in vitro*	W	B	(18)
2007	Biological characterization of a clinical and an environmental isolate of *Acanthamoeba polyphaga*: analysis of relevant parameters to decode pathogenicity	W	A	(19)
2007	Diagnosis of *Acanthamoeba* corneal Infection by impression cytology: Case report [Citologia de impressão no diagnóstico de infecção corneana por *Acanthamoeba*: Relato de caso]	C	A	(20)
2007	Disseminated *Balamuthia mandrillaris* amoeba infection in an AIDS patient from Brazil	C	B	(21)
2007	Isolation and identification of potentially pathogenic free-living amoebae in samples from environments in a public hospital in the city of Porto Alegre, Rio Grande do Sul	E	A	(22)
2008	*Acanthamoeba* spp. and bacterial contamination in contact lens storage cases and the relationship to user profiles	E	A	(23)
2008	*Acanthamoeba* spp. cysts storage in filter paper	W	A	(24)
2008	Corneal graft survival after therapeutic keratoplasty for *Acanthamoeba* keratitis	C	A	(25)
2008	Growth, encystment and survival of *Acanthamoeba castellanii* grasing on different bacteria	W	A	(26)
2008	*In vitro* evaluation of the amebicidal activity of *Pterocaulon* polystachyum (*Asteraceae*) against trophozoites of *Acanthamoeba castellanii*	W	A	(27)
2009	*Acanthamoeba* interaction with extracellular matrix glycoproteins: Biological and biochemical characterization and Role in Cytotoxicity and Invasiveness	W	A	(28)
2009	*Acanthamoeba* spp. in urine of critically ill patients	C	A	(29)
2009	Differential effects of alpha-helical and beta-hairpin antimicrobial peptides against *Acanthamoeba castellanii*	W	A	(30)
2009	Elastase secretion in *Acanthamoeba polyphaga*	W	A	(31)
2009	Genotyping, physiological features and proteolytic activities of a potentially pathogenic *Acanthamoeba* sp. isolated from tap water in Brazil	E	A	(32)
2009	Potentially pathogenic *Acanthamoeba* in swimming pools: A survey in the southern Brazilian city of Porto Alegre	E	A	(33)
2009	Prevalence of potentially pathogenic free-living amoebae from *Acanthamoeba* and *Naegleria* Genera in non-hospital, public, internal environments from the city of Santos, Brazil	E	A,N	(34)
2009	Twenty Years of *Acanthamoeba* Keratitis	C	A	(35)
2010	Characterization of *Acanthamoeba* isolates from dust of a public hospital in Curitiba, Paraná, Brazil	E	A	(36)
2010	Granulomatous Amoebic Meningoencephalitis in an Immunocompetent Patient	C	B	(37)

Year	Title	Category[a]	Target FLA[b]	#[c]
2010	*In vitro* amoebicidal activity of S-nitrosoglutathione and S-nitroso-N- acetylcysteine against trophozoites of *Acanthamoeba castellanii*	W	A	(38)
2010	Potentially Pathogenic *Acanthamoeba* Isolated from a Hospital in Brazil	E	A	(39)
2010	Susceptibility of *Aedes aegypti* (Diptera: Culicidae) to *Acanthamoeba polyphaga* (Sarcomastigophora: Acanthamoebidae)	W	A	(40)
2010	The fine structure of the *Acanthamoeba polyphaga* cyst wall	W	A	(41)
2011	*Acanthamoeba* T3, T4 and T5 in swimming-pool waters from Southern Brazil	W	A	(42)
2011	Amoebicidal activity and chemical composition of *Pterocaulon polystachyum* (Asteraceae) essential oil	W	A	(43)
2011	Assessing efficacy of combined riboflavin and UV-A light (365 nm) treatment of *Acanthamoeba* trophozoites	C	A	(44)
2011	Bacteriocin-like substance from *Bacillus amyloliquefaciens* shows remarkable inhibition of *Acanthamoeba polyphaga*	W	A	(45)
2011	Prevalence of *Acanthamoeba* from tap water in Rio Grande do Sul, Brazil	E	A	(46)
2011	Serine-like proteolytic enzymes correlated with differential pathogenicity in patients with acute *Acanthamoeba* keratitis	C	A	(47)
2012	A Rapid and Reliable Method for the Clonal Isolation of *Acanthamoeba* from Environmental Samples	E	A	(48)
2012	Chemical composition and amoebicidal activity of *Croton* p*allidulus*, *Croton* e*ricoides*, and *Croton isabelli* (Euphorbiaceae) essential oils	W	A	(49)
2012	Chemical composition and amoebicidal activity of *Piper hispidinervum* (Piperaceae) essential oil	W	A	(50)
2012	Meningoencephalitis caused by *Naegleria fowleri* in cattle of northeast Brazil	C	N	(51)
2012	Occurrence and characterization of *Acanthamoeba* similar to genotypes T4, T5, and T2/T6 isolated from environmental sources in Brasília, Federal District, Brazil	E	A	(52)
2012	Prevalence of *Acanthamoeba* spp. (Sarcomastigophora: Acanthamoebidae) in wild populations of *Aedes aegypti* (Diptera: Culicidae)	E	A	(53)
2012	Revisiting the *Acanthamoeba* species that form star-shaped cysts (genotypes T7, T8, T9, and T17): Characterization of seven new Brazilian environmental isolates and phylogenetic inferences	E	A	(54)
2013	Amoebicidal activity of phytosynthesised silver nanoparticles and their *in vitro* cytotoxicity to human cells	W	A	(55)
2013	Antimicrobial action of biguanides on the viability of *Acanthamoeba* cysts and assessment of cell toxicity	C	A	(56)
2013	*In vitro* effect of *Acanthospermum australe* (Asteraceae) extracts on *Acanthamoeba polyphaga* trophozoites [Efeito *in vitro* de extratos de *Acanthospermum australe* (Asteraceae) sobre trofozoitos de *Acanthamoeba polyphaga*]	W	A	(57)
2013	Infection in a rat model reactivates attenuated virulence after long-term axenic culture of *Acanthamoeba* spp	W	A	(58)
2013	Isolation and genotyping of free-living environmental isolates of *Acanthamoeba* spp. from bromeliads in Southern Brazil	E	A	(59)
2013	Morphological, genotypic, and physiological characterization of *Acanthamoeba* isolates from keratitis patients and the domestic environment in Vitoria, Espírito Santo, Brazil	C	A	(60)
2013	Physiological, morphological, and immunochemical parameters used for the characterization of clinical and environmental isolates of *Acanthamoeba*	E	A	(61)
2013	Selenocysteine biosynthesis and insertion machinery in *Naegleria gruberi*	W	N	(62)
2013	Susceptibility of *Acanthamoeba* to multipurpose lens-cleaning solutions	W	A	(63)
2014	Characterization of isolates of *Acanthamoeba* from the nasal mucosa and cutaneous lesions of dogs	C	A	(64)
2014	Proteomic profiling of the infective trophozoite stage of *Acanthamoeba polyphaga*	W	A	(65)
2015	A method for microbial decontamination of *Acanthamoeba* cultures using the peritoneal cavity of mice	W	A	(66)
2015	*Acanthamoeba* misidentification and multiple labels: redefining genotypes T16, T19, and T20 and proposal for *Acanthamoeba micheli* sp. nov. (genotype T19)	W	A	(67)
2015	*Acanthamoeba polyphaga* mimivirus prevents amoebal encystment-mediating serine proteinase expression and circumvents cell encystment	W	A	(68)
2015	*Acanthamoeba* T4, T5 and T11 Isolated From Mineral Water Bottles in Southern Brazil	E	A	(69)
2015	Cytotoxic activity and degradation patterns of structural proteins by corneal isolates of *Acanthamoeba* spp	W	A	(70)
2015	Identification of *Paenibacillus* as a Symbiont in *Acanthamoeba*	E	A	(71)
2015	Identification of pseudomonas spp. as amoeba-resistant microorganisms in isolates of *Acanthamoeba*	W	A	(72)
2015	Modulation of the expression of mimivirus-encoded translation-related genes in response to nutrient availability during *Acanthamoeba castellanii* infection	W	A	(73)
2015	Systemic acanthamoebiasis associated with canine distemper in dogs in the semiarid region of Paraíba, Brazil	C	A	(74)
2016	*Acanthamoeba* and *Fusarium* interactions: A possible problem in keratitis	W	A	(75)
2016	*Acanthamoeba* and mimivirus interactions: The role of amoebal encystment and the expansion of the 'Cheshire Cat' theory	R	A	(76)

Year	Title	Category[a]	Target FLA[b]	#[c]
2016	*Acanthamoeba* spp. as a universal host for pathogenic microorganisms: One bridge from environment to host virulence	R	A	(77)
2016	Amebicidal activity of the essential oils of Lippia spp. (Verbenaceae) against *Acanthamoeba polyphaga* trophozoites	W	A	(78)
2016	Crystallisation and Crystallographic Analyses of Triose phosphateIsomerase from *Naegleria gruberi*	W	N	(79)
2016	Detection and quantification of human Adenovirus genomes in *Acanthamoeba* isolated from swimming pools	E	A	(80)
2016	Evaluation of the immunodiagnostic potential of a recombinant surface protein domain from *Acanthamoeba castellanii*	W	A	(81)
2016	Experimental infection of T4 *Acanthamoeba* genotype determines the pathogenic potential	W	A	(82)
2016	Expression, purification, enzymatic characterization and crystallization of glyceraldehyde-3-phosphate dehydrogenase from *Naegleria gruberi*, the first one from phylum Percolozoa	W	N	(83)
2016	Isolation and identification of *Acanthamoeba* spp. from thermal swimming pools and spas in Southern Brazil	E	A	(84)
2016	Photodynamic inactivation of *Acanthamoeba polyphaga* with curcuminoids:an *in vitro* study	W	A	(85)
2016	Uncovering Cryptic Diversity in Two Amoebozoan Species Using Complete Mitochondrial Genome Sequences	W	A, W	(86)
2017	Anaerobic Metabolism in T4 *Acanthamoeba* Genotype	W	A	(87)
2017	Interaction Between Methicillin-Resistant Staphylococcus aureus (MRSA) and *Acanthamoeba polyphaga*	W	A	(88)
2017	Modulation of zinc homeostasis in *Acanthamoeba castellanii* as a possible antifungal strategy against *Cryptococcus gattii*	W	A	(89)
2017	Molecular diagnosis of *Acanthamoeba* keratitis: evaluation in rat model and application in suspected human cases	C	A	(90)
2017	Nanoemulsions Containing a Coumarin-Rich Extract from *Pterocaulon balansae* (Asteraceae) for the Treatment of Ocular *Acanthamoeba* keratitis	W	A	(91)
2017	Occurrence of Infected Free-Living Amoebae in Cooling Towers of Southern Brazil	E	A,N,W	(92)
2017	Sensitivity of Enzymatic Toxins from Corneal Isolate of *Acanthamoeba* Protozoan to Physicochemical Parameters	C	A	(93)
2017	siRNA-loaded liposomes: Inhibition of encystment of *Acanthamoeba* and toxicity on the eye surface	W	A	(94)
2017	Therapeutic agents and biocides for ocular infections by free-living amoebae of *Acanthamoeba* genus	R	A	(95)
2018	*Acanthamoeba* keratitis in patients wearing scleral contact lenses	C	A	(96)
2018	*Acanthamoeba* keratitis in Porto Alegre (southern Brazil): 28 cases and risk factors	C	A	(97)
2018	*Acanthamoeba* of three morphological groups and distinct genotypes exhibit variable and weakly inter-related physiological properties	E	A	(98)
2018	Comparative proteomic analysis of soluble and surface-enriched proteins from *Acanthamoeba* castellanii trophozoites	W	A	(99)
2018	Contact lens-related polymicrobial keratitis: *Acanthamoeba* spp. genotype T4 and *Candida albicans*	C	A	(100)
2018	Extracellular vesicles and vesicle-free secretome of the protozoa *Acanthamoeba castellanii* under homeostasis and nutritional stress and their damaging potential to host cells	W	A	(101)
2018	Structural studies of glyceraldehyde-3-phosphate dehydrogenase from *Naegleria gruberi*, the first one from phylum Percolozoa	S	N	(102)
2018	The first *Acanthamoeba* keratitis case in the midwest region of Brazil: Diagnosis, genotyping of the parasite and disease outcome	C	A	(103)
2018	The therapeutic strategies against *Naegleria fowleri*	R	N	(104)
2018	Virulent T4 *Acanthamoeba* causing keratitis in a patient after swimming while wearing contact lenses in Southern Brazil	C	A	(105)
2019	Box Behnken design of siRNA-loaded liposomes for the treatment of a murine model of ocular keratitis caused by *Acanthamoeba*	W	A	(106)
2019	Extracellular vesicles from the protozoa *Acanthamoeba castellanii*: Their role in pathogenesis, environmental adaptation and potential applications	R	A	(107)
2019	Genomic Signatures Among *Acanthamoeba polyphaga* Entoorganisms Unveil Evidence of Coevolution	S	A	(108)
2019	Intra-amoebic localisation of *Arcobacter butzleri* as an endocytobiont of *Acanthamoeba castellanii*	W	A	(19)
2019	Meningoencephalitis due to *Naegleria fowleri* in cattle in southern Brazil [Meningoencefalite por *Naegleria fowleri* em um bovino no sul do Brasil]	C	N	(110)
2019	Recovery of an *Acanthamoeba* strain with two group I introns in the nuclear 18S rRNA gene	W	A	(111)
2019	Unravelling the interactions of the environmental host *Acanthamoeba castellanii* with fungi through the recognition by mannose-binding proteins	W	A	(112)

Year	Title	Category[a]	Target FLA[b]	#[c]
2020	*Acanthamoeba castellanii* as an alternative interaction model for the dermatophyte *Trichophyton rubrum*	W	A	(113)
2020	*Acanthamoeba castellanii* phosphate transporter (AcPHS) is important to maintain inorganic phosphate influx and is related to trophozoite metabolic processes	W	A	(114)
2020	*Acanthamoeba* spp. monoclonal antibody against a CPA2 transporter: a promising molecular tool for acanthamoebiasis diagnosis and encystment study	W	A	(115)
2020	Extracellular protease profile of *Acanthamoeba* after prolonged axenic culture and after interaction with MDCK cells	W	A	(116)
2020	Free-living amoebae and its relationship with air quality in hospital environments: Isolation and characterization of *Acanthamoeba* spp. from air-conditioning system	E	A	(117)
2020	Identification of T3 and T4 Genotypes of *Acanthamoeba* sp. in Dust Samples Isolated from Air Conditioning Equipment of Public Hospital of Ituiutaba-MG	E	A, B	(118)
2020	*In vitro* amoebicidal activity of imidazolium salts against trophozoites	W	A	(119)
2020	Isolation of *Naegleria* spp. From a Brazilian water source	E	N,W	(120)
	**AUXILIARY**			
2013	Distribution of infectious keratitis in a tertiary hospital in Brazil	C	A	(121)
2008	Contact lens-associated microbial keratitis	C	A	(122)
2017	Results with the Boston Type I keratoprosthesis after *Acanthamoeba* keratitis	C	A	(123)
2017	Epidemiological profile of infectious keratitis	C	A	(124)
2015	Degeneration and Regeneration of Sub basal Corneal Nerves after Infectious Keratitis: A Longitudinal *in Vivo* Confocal Microscopy Study	C	A	(125)
2018	Central nervous system disorders diagnosed in dogs	C	A, N, B	(126)
2013	Yaravirus: A novel 80-nm virus infecting *Acanthamoeba castellanii*	E	A	(127)
2020	Occurrence of parasites in salads in restaurants in Aparecida de Goiâniaia, Goias, Brazil	E	A	(128)
2018	Genotypic characterization and assessment of infectivity of human waterborne pathogens recovered from oysters and estuarine waters in Brazil	E	A	(129)
2020	Isolation and genomic characterization of a new mimivirus of lineage B from a Brazilian river	E	A	(130)
2019	A hidden battle in the dirt: Soil amoebae interactions with *Paracoccidioides* spp	E	A, W	(131)
2016	Giants among larges: How gigantism impacts giant virus entry into amoebae	R	A	(132)
2011	Diffusion Imaging in Brain Infections	R	A	(133)
2014	*Acanthamoeba polyphaga* mimivirus and other giant viruses: An open field to outstanding discoveries	R	A	(134)
2020	The Sexual Ancestor of all Eukaryotes: A Defense of the " Meiosis Toolkit " A Rigorous Survey Supports the Obligate Link between Meiosis Machinery and Sexual Recombination	R	A	(135)
2019	Discovery and further studies on giant viruses at the IHU mediterranee infection that modified the perception of the virosphere	R	A	(136)
2016	Editorial overview: The megaviromes	R	A	(137)
2013	Light based anti-infectives: Ultraviolet C irradiation, photodynamic therapy, blue light, and beyond	R	A	(138)
2019	Emerging infectious diseases with cutaneous manifestations Fungal, helminthic, protozoan and ectoparasitic infections	R	B	(139)
2012	Co-infection of HIV and tropical infectious agents that affect the nervous system	R	A,N,B	(140)
2017	Emerging Infections and Pertinent Infections Related to Travel for Patients with Primary Immunodeficiencies	R	A,N,S,B	(141)
2011	A morphological approach to the diagnosis of protozoal infections of the central nervous system	R	A,N,S,B	(142)
2019	Giant virus vs amoeba: Fight for supremacy	R	A, W	(143)
2019	Editorial: Large and giant DNA viruses	R	A, W	(144)
2019	Guarani virophage, a new sputnik-like isolate from a Brazilian lake	W	A	(145)
2019	Microscopic Analysis of the Tupanvirus Cycle in *Vermamoeba vermiformis*	W	A	(146)
2018	Lack of evidence of mimivirus replication in human PBMCs	W	A	(147)
2017	Filling Knowledge Gaps for Mimivirus Entry, Uncoating, and Morphogenesis	W	A	(148)
2013	Identification and functional characterization of K+ transporter sencoded by *Legionella pneumophila* kup genes	W	A	(149)
2019	Transcriptional analysis of flagellar and putative virulence genes of *Arcobacter butzleri* as an endocytobiont of *Acanthamoeba castellanii*	W	A	(150)
2014	*Acanthamoeba polyphaga* mimivirus stability in environmental and clinical substrates: Implications for virus detection and isolation	W	A	(151)

Year	Title	Category[a]	Target FLA[b]	#[c]
2020	Antifungal and antiprotozoal green amino acid-based rhamnolipids: Mode of action, antibiofilm efficiency and selective activity against resistant *Candida* spp. strains and *Acanthamoeba castellanii*	W	A	(152)
2020	Analysis of a Marseillevirus Transcriptome Reveals Temporal Gene Expression Profile and Host Transcriptional Shift	W	A	(153)
2020	The interaction between *Sporothrix schenckii* sensu stricto and *Sporothrix brasiliensis* with *Acanthamoeba castellanii*	W	A	(154)
2019	New Isolates of Pandoraviruses: Contribution to the Study of Replication Cycle Steps	W	A	(155)
2019	Role of the R349 gene and its repeats in the mimivire defense system	W	A	(156)
2018	Rio Negro virophage: Sequencing of the near complete genome and transmission electron microscopy of viral factories and particles	W	A	(157)
2018	Analyses of the Kroon virus major capsid gene and its transcript highlight a distinct pattern of gene evolution and splicing among mimiviruses	W	A	(158)
2017	The investigation of promoter sequences of marseilleviruses highlights a remarkable abundance of the AAATATTT motif in intergenic regions	W	A	(159)
2017	Analysis of multiple components involved in the interaction between *Cryptococcus neoformans* and *Acanthamoeba castellanii*	W	A	(160)
2017	Microscopic characterization of the Brazilian giant samba virus	W	A	(161)
2016	The large marseillevirus explores different entry pathways by forming giant infectious vesicles	W	A	(162)
2016	Different Photoresponses of Microorganisms: From Bioinhibition to Biostimulation	W	A	(163)
2016	A Brazilian marseillevirus is the founding member of a lineage in family marseilleviridae	W	A	(164)
2015	Mimivirus fibrils are important for viral attachment to the microbial world by a diverse glycoside interaction repertoire	W	A	(165)
2015	*Niemeyer* virus: A new mimivirus group A isolate harboring a set of duplicated aminoacyl-tRNA synthetase genes	W	A	(166)
2014	Samba virus: A novel mimivirus from a giant rain forest, the Brazilian Amazon	W	A	(167)
2014	Amoebas as mimivirus bunkers: Increased resistance to UV light, heat and chemical biocides when viruses are carried by amoeba hosts	W	A	(168)
2014	A resourceful giant: APMV is able to interfere with the human type I interferon system	W	A	(169)
2013	Active metabolites produced by *Penicillium chrysogenum* IFL1 growing on agro-industrial residues	W	A	(170)
2013	The Transcriptional Response of *Cryptococcus neoformans* to Ingestion by *Acanthamoeba castellanii* and Macrophages Provides Insights into the Evolutionary Adaptation to the Mammalian Host	W	A	(171)
2012	Virucidal activity of chemical biocides against mimivirus, a putative pneumonia agent	W	A	(172)
2019	In-depth analysis of the replication cycle of Orpheovirus	W	W	(173)
2019	Trapping the Enemy: Vermamoeba vermiformis Circumvents *Faustovirus mariensis* Dissemination by Enclosing Viral Progeny inside Cysts	W	A, W	(174)
2020	Virus goes viral: An educational kit for virology classes	W	A,W	(175)
2018	Tailed giant Tupanvirus possesses the most complete translational apparatus of the known virosphere	W	A,W	(176)
2015	Isolation of new Brazilian giant viruses from environmental samples using a panel of protozoa	W	A,W	(177)
2014	Growing a giant: Evaluation of the virological parameters for Mimivirus production	W	A	(178)

a: category: Wet-bench(W); Dry-bench(S); Review(R); Environmental(E); Clinical(C). *b*: Target FLA: *Acanthamoeba* (A), *Vermamoeba*(V), *Naegleria* (N), *Balamuthia* (B). *c*: the numbers do not correspond to the reference numbers in the original paper.

TABLE II

Key words (I), acronyms (II) and inclusion/exclusion criteria (III) used to search in the databases

I. KEY WORDS		
#	Key word	Synonyms/misspelling
1	naegleria	OR negleria
2	free-living amoeba	free living amoeba OR freeliving amoeba OR free-living amoebic OR freeliving amoebic OR free-living amebic OR free living amoebic OR free living amebic
3	amebiasis	OR amoebiasis
4	amoebic infection	OR amebic infection
5	primary amoebic meningoencephalitis	primary meningoencephalitis OR primary amebic meningoencephalitis OR amoebic meningoencephalitis OR amebic meningoencephalits
6	amoebic encephalitis	OR amebic encephalitis
7	granulomatous amoebic encephalitis	granulomatous encephalitis OR granulomatous amebic encephalitis OR granulomatus amoebic encephalitis
8	Acanthamoeba	OR Acantamoeba OR Acanthameoba OR Acanthamebiasis OR Acanthameba
9	amoebic keratitis	OR amebic keratitis OR amoeba keratitis OR Acanthamoeba keratitis
10	Sappinia	OR Sappinea OR Sapinia OR sappinia amoebic encephalitis
11	Balamuthia	OR Ballamuthia OR Balamuthias OR Balamuthiasis
12	Vermamoeba	OR Vermameba
13	Hartmannella	OR Hartmanella
14	Brazil	OR brasil

II. ACRONYMS		
**Key word #**	**Web of Science**	**Outcome (# of papers)**
#1	TI = (naegleria OR negleria) OR AB = (naegleria OR negleria) OR AK = (naegleria OR negleria)	1432
#14	AD =(Brazil OR brasil)	1101096
#1 AND #14	(#1 AND #14)	14
**Key word #**	**PubMed**	**Outcome (# of papers)**
#1	naegleria[Title/Abstract] OR negleria[Title/Abstract]	1337
#14	brazil[Affiliation]	428131
#1 AND #14	naegleria[Title/Abstract] OR negleria[Title/Abstract] AND brazil[Affiliation]	11
**Key word #**	**Scopus**	**Outcome (# of papers)**
#1	TITLE-ABS-KEY (naegleria OR negleria)	1889
#14	AFFIL (brazil OR brasil)	1206542
#1 AND #14	TITLE-ABS-KEY (naegleria OR negleria) AND AFFIL (brazil OR brasil)	19

III. CRITERIA	
**Inclusion criteria**	**Exclusion criteria**
1. To study any pathogenic free-living amoeba genera	1. Dissertations and thesis
2. Brazilian scope	2. Paper published in non-indexed journals
3. To be authored by Brazilian researchers	3. Not related to free-living amoeba
4. Dated from 2020 or earlier	4. Letter, erratum, book chapter, protocol, conference abstract
	5. Affiliations exclusively with foreign institutions (no participation of Brazilian researchers)
	6. Experimental research did not accomplish in Brazil
	7. Dated from 2021

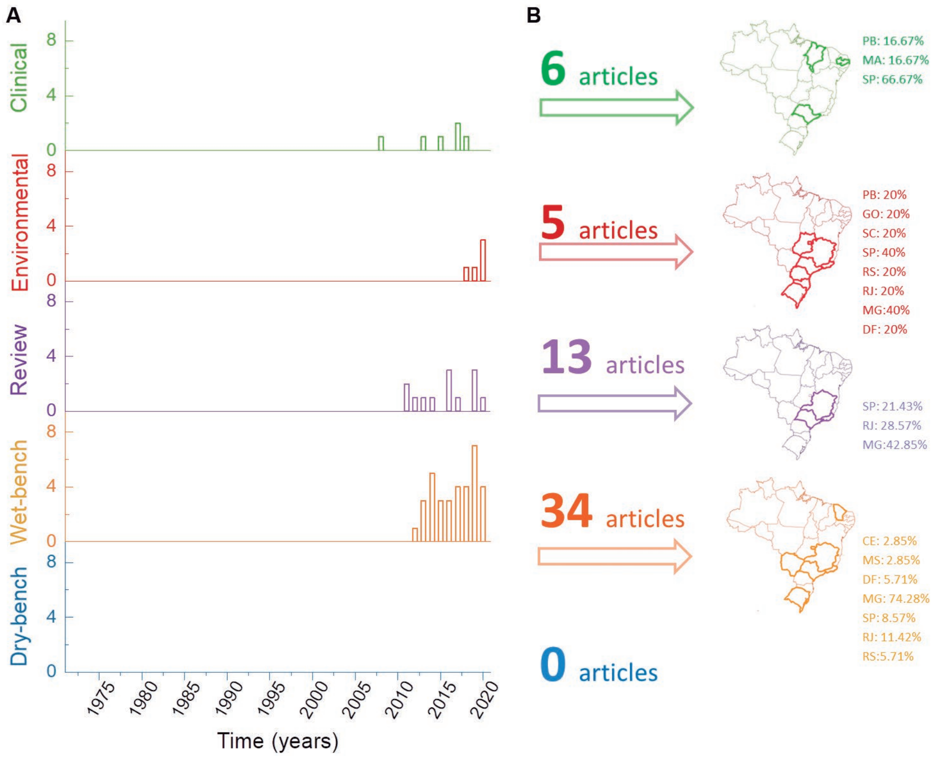

Discriminative free-living amoeba (FLA) literature in Brazil tagged as AUXILIARY, clustered into five categories: clinical, environmental, review, wet, and dry bench. (A) Stacked bar graph comparing the number of papers published per year per category. (B) Territorial scope depicting the percentage of papers per Federative Unit.

REFERENCES

1. Marzzoco A, Colli W. Isolation of nuclei and characterization of nuclear DNA of *Acanthamoeba castellanii*. Biochim Biophys Acta. 1974;374(3):292–303.

2. Marzzoco A, Colli W. Characterization of the genome of the small free-living amoeba *Acanthamoeba castellanii*. Biochim Biophys Acta. 1975;395(4):525–34.

3. Campos R, Gomes MCO, Prigenzi LS, Stecca J. Meningencefalite por ameba de vida livre apresentação do primeiro caso latino-americano. Rev Inst Med Trop São Paulo. 1977;19(5):349–51.

4. Salles-Gomes JR CE, Barbosa ER., Nobrega JP., Scaff M., Spina--França A. Primary amebic meningoencephalomyelitis. Report of a case. Arq Neuropsiquiatr. 1978;36(2):139–42.

5. de Moura H, Salazar HC, Fernandes O, Lisboa DC, de Carvalho FG. Free-living amoebae in human intestine: Evidence of parasitism. Rev Inst Med Trop Sao Paulo. 1985;27(3):150–6.

6. Salazar HC, Moura H, Fernandez O, Peralta JM. Isolation of *Naegleria fowleri* from a lake in the city of Rio de Janeiro. Trans R Soc Trop Med Hyg. 1986;80(2):348–9.

7. de Freitas D, Foronda AS, Sat ÉH, Júnior RB, Uras R. *Acanthamoeba culbertsoni*: sensibilidade aos diferentes métodos de desinfecção de lentes de contacto hidrofílicas. Arq Bras Oftal. 1987;1(3):2–6.

8. Moura H, Wallace S, Visvesvara GS. *Acanthamoeba healyi* N. sp. and the isoenzyme and immunoblot profiles of *Acanthamoeba* spp., groups 1 and 3. J Protozool. 1992;39(5):573–83.

9. Chimelli L, Hahn MD, Scaravilli F, Sara Wallace, Visvesvara GS. Granulomatous amoebic encephalitis due to leptomyxid amoebae : report of the first Brazilian case. Trans R Soc Trop Med Hyg. 1992;86(6):635.

10. Alvarenga LS, de Freitas D, Hofling-Lima AL. Ceratite por *Acanthamoeba*. Arq Bras Oftal. 2000;63(1):155–9.

11. Alfieri SC, Correia CEB, Motegi SA, Pral EMF. Proteinase activities in total extracts and in medium conditioned by *Acanthamoeba polyhaga* trophozoites. J Parasitol. 2000;86(2):220–7.

12. Alves JM, Gusmão CX, Teixeira MMG, de Freitas D, Foronda AS, Affonso HT. Random amplified polymorphic DNA profiles as a tool for the characterization of Brazilian keratitis isolates of the genus *Acanthamoeba*. Braz J Med Biol Res. 2000;33(1):19–26.

13. Forseto AS, Nosé W. Diagnosis of *Acanthamoeba* keratitis with confocal microscopy. Vol. 62, Rev Bras de Oftalmol. 2003. p. 220–8.

14. da Silva MA, da Rosa JA. Isolation of potencially pathogenic free-living amoebas in hospital dust. Rev Saude Publica. 2003;37(2):242–6.

15. Nakano E, Oliveira M, Portellinha W, de Freitas D, Nakano K. Confocal microscopy in early diagnosis of *Acanthamoeba* keratitis. J Refract Surg. 2004;20(5):S737-741.

16. Ruthes ACC, Wahab S, Wahab N, Moreira H, Moreira L. Conjunctivitis presumably due to *Acanthamoeba*. Arq Bras Oftal. 2004;67(6):897–900.

17. da Rocha-Azevedo B, Menezes GC, Costa e Silva-Filho F. The interaction between *Acanthamoeba polyphaga* and human osteoblastic cells *in vitro*. Microb Pathog. 2006;40(1):8–14.

18. da Rocha-Azevedo B, Jamerson M, Cabral GA, Costa e Silva-Filho F, Marciano-Cabral F. The interaction between the amoeba *Balamuthia mandrillaris* and extracellular matrix glycoproteins *in vitro*. Parasitology. 2007;134((Pt 1)):51–8.

19. da Rocha-Azevedo B, Costa e Silva-Filho F. Biological characterization of a clinical and an environmental isolate of *Acanthamoeba polyphaga*: analysis of relevant parameters to decode pathogenicity. Arch Mircobiol. 2007;188(5):441–9.

20. Barros JN, Mascaro VLD, Lowen M, Martins MC, Foronda A. Diagnosis of *Acanthamoeba* corneal infection by impression cytology: case report. Arq Bras Oftal. 2007;70(2):343–6.

21. Silva-vergara ML, Colombo ER da C, Vissotto EDF, Silva ACAL, Chica JEL, Etchebehere RM, et al. Disseminated *Balamuthia mandrillaris* amoeba infection in an AIDS patient from Brazil. Am J Trop Med Hyg. 2007;77(6):1096–8.

22. Carlesso AM, Simonetti AB, Artuso GL, Rott MB. Isolation and identification of potentially pathogenic free-living amoebae in samples from environments in a public hospital in the City of Porto Alegre, Rio Grande do Sul. Rev Soc Bras Med Tro. 2007;40(3):316–20.

23. Pens CJ, Costa M da, Fadanelli C, Caumo K, Rott MB. *Acanthamoeba* spp . and bacterial contamination in contact lens storage cases and the relationship to user profiles. Parasitol Res. 2008;103(6):1241–5.

24. Pens CJ, Rott MB. *Acanthamoeba* spp. cysts storage in filter paper. Parasitol Res. 2008;103(5):1229–30.

25. Kashiwabuchi RT, de Freitas D, Alvarenga LS, Vieira L, Contarini P, Sato E, et al. Corneal graft survival after therapeutic keratoplasty for *Acanthamoeba* keratitis. Acta Ophthalmol. 2008;86(6):666–9.

26. Moraes J, Alfieri SC. Growth, encystment and survival of *Acanthamoeba castellanii* grazing on different bacteria. FEMS Microbiol Ecol. 2008;66(2):221–9.

27. Ródio C, da Rocha Vianna D, Kowalski KP, Panatiere LF, von Poser G, Rott MB. *In vitro* evaluation of the amebicidal activity of *Pterocaulon polystachyum* (Asteraceae) against trophozoites of *Acanthamoeba castellanii*. Parasitol Res. 2008;104(1):191–4.

28. da Rocha-Azevedo B, Jamerson M, Cabral GA, Costa e Silva-Filho F, Marciano-Cabral F. *Acanthamoeba* interaction with extracellular matrix glycoproteins: biological and biochemical characterization and role in cytotoxicity and invasiveness. J Eukaryot Microbiol. 2009;56(3):270–8.

29. Santos LC, Oliveira MS, Lobo RD, Higashino HR, Costa SF, Van Der Heijden IM, et al. *Acanthamoeba* spp. in urine of critically ill patients. Emerg Infect Dis. 2009;15(7):1144–6.

30. Sacramento RS, Martins RM, Miranda A, Dobroff ASS, Daffre S, Foronda AS, et al. Differential effects of alpha-helical and beta--hairpin antimicrobial peptides against *Acanthamoeba castellanii*. Parasitology. 2009;136(8):813–21.

31. Ferreira GA, Magliano ACM, Pral EMF, Alfieri SC. Elastase secretion in *Acanthamoeba polyphaga*. Acta Trop. 2009;112(2):156–63.

32. Magliano ACM, da Silva FM, Teixeira MMG, Alfieri SC. Genotyping, physiological features and proteolytic activities of a potentially pathogenic *Acanthamoeba* sp. isolated from tap water in Brazil. Exp Parasitol. 2009;123(3):231–5.

33. Caumo K, Frasson AP, Pens CJ, Panatieri LF, Frazzon APG, Rott MB. Potentially pathogenic *Acanthamoeba* in swimming pools: a survey in the southern Brazilian city of Porto Alegre. Ann Trop Med Parasitol. 2009;103(6):477–85.

34. Teixeira LH, Rocha S, Pinto RMF, Caseiro MM, Costa SOP. Prevalence of potentially pathogenic free-living amoebae from *Acanthamoeba* and *Naegleria* genera in non-hospital, public, internal environments from the city of Santos, Brazil. Brazilian J Infect Dis. 2009;13(6):395–7.

35. Carvalho FRS, Foronda AS, Mannis MJ, Hofling-Lima AL, Belfort RJr, de Freitas D. Twenty years of *Acanthamoeba* keratitis. Cornea. 2009;28(5):516–9.

36. Costa AO, Castro EA, Ferreira GA, Furst C, Crozeta MA, Thomas-Soccol V. Characterization of *Acanthamoeba* isolates from dust of a public hospital in Curitiba, Paraná, Brazil. J Eukaryot Microbiol. 2010;57(1):70–5.

37. Silva RA, Araújo SDA, Avellar IFDF, Pittella JEH, De Oliveira JT, Christo PP. Granulomatous amoebic meningoencephalitis in an immunocompetent patient. Arch Neurol. 2010;67(12):1516–20.

38. Cariello AJ, Souza GFPe, Foronda AS, Yu MCZ, Hofling-Lima AL, Oliveira MG. *In vitro* amoebicidal activity of S-nitrosoglutathione and S-nitroso-N-acetylcysteine against trophozoites of *Acanthamoeba castellanii*. J Antimicrob Chemother. 2010;65(3):588–91.

39. Carlesso AM, Artuso GL, Caumo K, Rott MB. Potentially pathogenic *Acanthamoeba* isolated from a hospital in Brazil. Curr Microbiol. 2010;60(3):185–90.

40. Rott M, Caumo K, Sauter I, Eckert J, da Rosa L, da Silva O. Susceptibility of *Aedes aegypti* (Diptera:Culicidae) to *Acanthamoeba polyphaga* (Sarcomastigophora:Acanthamoebidae). Parasitol Res. 2010;107(1):195–8.

41. Lemgruber L, Lupetti P, De Souza W, Vommaro RC,da Rocha-Azevedo B. The fine structure of the *Acanthamoeba polyphaga* cyst wall. FEMS Microbiol Lett. 2010;305(2):170–6.

42. Caumo K, Rott MB. *Acanthamoeba* T3, T4 and T5 in swimming-pool waters from Southern Brazil. Acta Trop. 2011;117(3):233–5.

43. Sauter IP, Santos JC, Apel MA, Cibulski SP, Roehe PM, von Poser GL, et al. Amebicidal activity and chemical composition of *Pterocaulon polystachyum* (Asteraceae) essential oil. Parasitol Res. 2011;109(5):1367–71.

44. Kashiwabuchi RT, Carvalho FRS, Khan YA, Freitas D De, Foronda AS, Hirai FE, et al. Assessing efficacy of combined riboflavin and UV-A Light (365 nm) treatment of *Acanthamoeba* trophozoites. Invest Ophthalmol Vis Sci. 2011;52(13):9333–8.

45. Benitez LB, Caumo K, Brandelli A, Rott MB. Bacteriocin-like substance from *Bacillus amyloliquefaciens* shows remarkable inhibition of *Acanthamoeba polyphaga*. Parasitol Res. 2011;108(3):687–91.

46. Winck MAT, Caumo K, Rott MB. Prevalence of *Acanthamoeba* from tap water in Rio Grande do Sul, Brazil. Curr Microbiol. 2011;63(5):464–9.

47. Carvalho FRDS, Carrijo-Carvalho LC, Chudzinski-Tavassi AM, Foronda AS, de Freitas D. Serine-like proteolytic enzymes correlated with differential pathogenicity in patients with acute *Acanthamoeba* keratitis. Clin Microbiol Infect. 2010;17(4):603–9.

48. Zanella J, da Costa SOP, Zacaria J, Echeverrigaray S. A rapid and reliable method for the clonal isolation of *Acanthamoeba* from environmental samples. Brazilian Arch Biol Technol. 2012;55(1):1–6.

49. Vunda SLL, Sauter IP, Cibulski SP, Roehe PM, Bordignon SAL, Rott MB, et al. Chemical composition and amoebicidal activity of *Croton pallidulus*, *Croton ericoides*, and *Croton isabelli* (Euphorbiaceae) essential oils. Parasitol Res. 2012;111(3):961–6.

50. Sauter IP, Rossa GE, Lucas AM, Cibulski SP, Roehe PM, Silva LAA, et al. Chemical composition and amoebicidal activity of *Piper hispidinervum* (Piperaceae) essential oil. Ind Crop Prod. 2012;40:292–5.

51. Pimentel LA, Dantas AFM, Uzal F, Riet-Correa F. Meningoencephalitis caused by *Naegleria fowleri* in cattle of northeast Brazil. Res Vet Sci. 2012;93(2):811–2.

52. Alves DSMM, Moraes AS, Nitz N, Oliveira MGC, Hecht MM, Gurgel-Gonçalves R, et al. Occurrence and characterization of *Acanthamoeba* similar to genotypes T4 , T5 , and T2 / T6 isolated from environmental sources in Brasília , Federal District , Brazil. Exp Parasitol. 2012;131(2):239–44.

53. Otta DA, Rott MB, Carlesso AM, Santos O. Prevalence of *Acanthamoeba* spp . (Sarcomastigophora : Acanthamoebidae) in wild populations of Aedes aegypti (Diptera : Culicidae). Parasitol Res. 2012;(111):2017–22.

54. Magliano ACM, Teixeira MMG, Alfieri SC. Revisiting the *Acanthamoeba* species that form star-shaped cysts (genotypes T7, T8, T9, and T17): characterization of seven new Brazilian environmental isolates and phylogenetic inferences. Parasitology. 2011;139(1):45–52.

55. Borase HP, Patil CD, Sauter IP, Rott MB, Patil S V. Amoebicidal activity of phytosynthesized silver nanoparticles and their *in vitro* cytotoxicity to human cells. FEMS Microbiol Lett. 2013;345(2):127–31.

56. Mafra CSP, Carrijo-Carvalho LC, Chudzinski-tavassi AM, Taguchi FMC, Foronda AS, Carvalho FR de S, et al. Antimicrobial action of biguanides on the viability of *Acanthamoeba* cysts and assessment of cell toxicity. Invest Ophthalmol Vis Sci. 2013;54(9):6363–72.

57. Castro LC, Sauter IP, Ethur EM, Kauffmann C, Dall´agnol R, Souza J, et al. *In vitro* effect of *Acanthospermum australe* (Asteraceae) extracts on *Acanthamoeba polyphaga* trophozoites. Rev Bras Pl Med. 2013;15(4):589–94.

58. Veríssimo CDM, Maschio VJ, Correa APF, Brandelli A, Rott MB. Infection in a rat model reactivates attenuated virulence after long-term axenic culture of *Acanthamoeba* spp. Mem Inst Oswaldo Cruz. 2013;108(7):832–5.

59. Landeli MF, Salton J, Caumo K, Broetto L, Rott MB. Isolation and genotyping of free-living environmental isolates of *Acanthamoeba* spp . from bromeliads in Southern Brazil. Exp Parasitol. 2013;134(3):290–4.

60. Duarte JL, Furst C, Klisiowicz DR, Klassen G, Costa AO. Morphological, genotypic, and physiological characterization of *Acanthamoeba* isolates from keratitis patients and the domestic environment in Vitoria, Espírito Santo, Brazil. Exp Parasitol. 2013;135(1):9–14.

61. Becker-Finco A, Costa AO, Silva SK, Ramada JS, Furst C, Stingher AE, et al. Physiological , morphological , and immunochemical parameters used for the characterization of clinical and environmental isolates of *Acanthamoeba*. Parasitology. 2013;140(3):396–405.

62. da Silva MTA, Caldas VEA, Costa FC, Silvestre DAMM, Thiemann OH. Selenocysteine biosynthesis and insertion machinery in *Naegleria gruberi*. Mol Biochem Parasitol. 2013;188(2):87–90.

63. de Aguiar APC, de Oliveira Silveira C, Todero Winck MA, Rott MB. Susceptibility of *Acanthamoeba* to multipurpose lens-cleaning solutions. Acta Parasitol. 2013;58(3):304–8.

64. Carlesso AM, Mentz MB, Machado MLS, Carvalho A, Nunes TET, Maschio VJ, et al. Characterization of isolates of *Acanthamoeba* from the nasal mucosa and cutaneous lesions of dogs. Curr Microbiol. 2014;68(6):702–7.

65. Caumo KS, Monteiro KM, Ott TR, Maschio VJ, Wagner G, Ferreira HB, et al. Proteomic profiling of the infective trophozoite stage of *Acanthamoeba polyphaga*. Acta Trop. 2014;140:166–72.

66. Alves DSMM, Gurgel-Gonçalves R, Albuquerque P, Cuba-Cuba CA, Muniz-Junqueira MI, Kuckelhaus SAS. A method for microbial decontamination of *Acanthamoeba* cultures using the peritoneal cavity of mice. Asian Pac J Trop Biomed. 2015;5(10):796–800.

67. Corsaro D, Walochnik J, Köhsler M, Rott, M.B. *Acanthamoeba* misidentification and multiple labels: redefining genotypes T16, T19, and T20 and proposal for *Acanthamoeba micheli* sp. nov. (genotype T19). Parasitol Res. 2015; 114: 2481–2490.

68. Boratto P, Albarnaz JD, Almeida GMDF, Botelho L, Fontes ACL, Costa AO, et al. *Acanthamoeba polyphaga* mimivirus prevents amoebal encystment-mediating serine proteinase expression and circumvents cell encystment. J Virol. 2015;89(5):2962–5.

69. Maschio VJ, Chies F, Carlesso AM, Carvalho A, Rosa SP, Van Der Sand ST, et al. *Acanthamoeba* T4, T5 and T11 isolated from mineral water bottles in Southern Brazil. Curr Microbiol. 2014;70(1):6–9.

70. Sant´ana VP, Carrijo-Carvalho LC, Foronda AS, Chudzinski--Tavassi AM, de Freitas D, Ramos F, et al. Cytotoxic activity and degradation patterns of structural proteins by corneal isolates of *Acanthamoeba* spp. Graefes Arch Clin Exp Ophtalmol. 2015;253(1):65–75.

71. Maschio VJ, Corção G, Bücker F, Caumo K, Rott MB. Identification of *Paenibacillus* as a symbiont in *Acanthamoeba*. Curr Microbiol. 2015;71(3):415–20.

72. Maschio VJ, Corção G, Rott MB. Identification of *Pseudomonas* spp . as amoeba-resistant microorganisms in isolates of *Acanthamoeba*. Rev Inst Med Trop Sao Palo. 2015;57(1):81–3.

73. Silva LCF, Almeida GMF, Assis FL, Albarnaz JD, Boratto PVM, Dornas FP, et al. Modulation of the expression of mimivirus-encoded translation-related genes in response to nutrient availability during *Acanthamoeba castellanii* infection. Front Microbiol. 2015;6(6):539.

74. Frade MTS, Melo LF, Pessoa CRM, Araújo JL, Fighera RA, Souza AP, et al. Systemic acanthamoebiasis associated with canine distemper in dogs in the semiarid region of Paraíba , Brazil. Pesq Vet Bras. 2015;35(2):160–4.

75. Nunes TET, Brazil NT, Fuentefria AM, Rott MB. *Acanthamoeba* and *Fusarium* interactions: a possible problem in keratitis. Acta Trop. 2016;157:102–7.

76. Silva LKDS, Boratto PVM, La Scola B, Bonjardim CA, Abrahão JS. *Acanthamoeba* and mimivirus interactions: the role of amoebal encystment and the expansion of the 'Cheshire Cat' theory. Curr Opin Microbiol. 2016;31:9–15.

77. Guimaraes AJ, Gomes KX, Cortines JR, Peralta JM, Peralta RHS. *Acanthamoeba* spp. as a universal host for pathogenic microorganisms: one bridge from environment to host virulence. Microbiol Res. 2016;193:30–8.

78. Santos IGA, Scher R, Rott MB, Menezes LR, Costa EV, Cavalcanti SCH, et al. Amoebicidal activity of the essential oils of *Lippia* spp. (Verbenaceae) against *Acanthamoeba* polyphaga trophozoites. Parasitol Res. 2016;115(2):535–40.

79. Penteado RF, Martini VP, Iulek J. Crystallization and crystallographic analyses of triosephosphate isomerase from *Naegleria gruberi*. Rev Virtual Quim. 2016;8(6):1835–41.

80. Staggemeier R, Arantes T, Caumo KS. Detection and quantification of human adenovirus genomes in *Acanthamoeba* isolated from swimming pools. An Acad Bras Cienc. 2016;88:635–41.

81. Sánchez AGC, Virginio VG, Maschio VJ, Ferreira HB, Rott MB. Evaluation of the immunodiagnostic potential of a recombinant surface protein domain from *Acanthamoeba castellanii*. Parasitology. 2021;143(12):1656–64.

82. Alves DSMM, Moraes AS, Alves LM, Gurgel-Gonçalves R, Lino Júnior RS, Cuba-Cuba CA, et al. Experimental infection of T4 *Acanthamoeba* genotype determines the pathogenic potential. Parasitol Res. 2016;115(9):3435–40.

83. Machado ATP, Silva M, Iulek J. Expression, purification, enzymatic characterization and crystallization of glyceraldehyde-3-phosphate dehydrogenase from *Naegleria gruberi*, the first one from phylum Percolozoa. Protein Expr Purif. 2016;127:125–30.

84. Fabres LF, Santos SPR, Benitez LB, Rott MB. Isolation and identification of *Acanthamoeba* spp . from thermal swimming pools and spas in Southern Brazil. Acta Parasitol. 2016;61(2):221–7.

85. Corrêa TQ, Geralde MC, Carvalho MT, Bagnato VS, Kurachi C, Souza CWO. Photodynamic inactivation of *Acanthamoeba polyphaga* with curcuminoids: an *in vitro* study. Opt Methods Tumor Treat Detect Mech Tech Photodyn Ther XXV. 2016;9694:1–7.

86. Fuciková K, Lahr DJG. Uncovering cryptic diversity in two amoebozoan species using complete mitochondrial genome sequences. J Eukaryot Microbiol. 2016;63(1):112–22.

87. Alves DSMM, Alves LM, Costa TL, Castro AM, Vinaud MC. Anaerobic metabolism in T4 *Acanthamoeba* genotype. Curr Microbiol. 2017;74(6):685–90.

88. Souza TK, Soares SS, Benitez LB, Rott MB. Interaction between methicillin-resistant Staphylococcus aureus (MRSA) and *Acanthamoeba polyphaga*. Curr Microbiol. 2017;74(5):541–9.

89. Ribeiro NS, Santos FM, Garcia AWA, Ferrareze PAG, Fabres LF, Schrank A, et al. Modulation of zinc homeostasis in *Acanthamoeba castellanii* as a possible antifungal strategy against *Cryptococcus gattii*. Front Microbiol. 2017;8(August):1–11.

90. Costa AO, Furst C, Rocha LO, Cirelli C, Cardoso CN, Neiva FS, et al. Molecular diagnosis of *Acanthamoeba* keratitis: evaluation in rat model and application in suspected human cases. Parasitol Res. 2017;116(4):1339–44.

91. Panatieri LF, Brazil NT, Faber K, Medeiros-Neves B, von Poser GL, Rott MB, et al. Nanoemulsions containing a coumarin-rich extract from *Pterocaulon balansae* (Asteraceae) for the treatment of ocular *Acanthamoeba* keratitis. AAPS PharmSciTech. 2017;18(3):721–8.

92. Soares SS, Souza TK, Berte FK, Cantarelli VV, Rott MB. Occurrence of infected free-living amoebae in cooling towers of southern Brazil. Curr Microbiol. 2017;74(12):1461–8.

93. Sant´ana VP, Foronda AS, Freitas D de, Carrijo-Carvalho LC, Carvalho FR de S. Sensitivity of enzymatic toxins from corneal isolate of *Acanthamoeba* protozoan to physicochemical parameters. Curr Microbiol. 2017;74(11):1316–23.

94. Faber K, Zorzi GK, Brazil NT, Rott MB, Teixeira HF. siRNA-loaded liposomes: inhibition of encystment of *Acanthamoeba* and toxicity on the eye surface. Chem Biol Drug Des. 2017;90(3):406–16.

95. Carrijo-Carvalho LC, Sant´ana VP, Foronda AS, de Freitas D, Ramos F, Carvalho DS. Therapeutic agents and biocides for ocular infections by free-living amoebae of *Acanthamoeba* genus. Surv Ophthalmol. 2016;62(2):203–18.

96. Sticca MP, Carrijo-carvalho LC, Silva IMB, Vieira LA, Souza LB, Junior RB, et al. *Acanthamoeba* keratitis in patients wearing scleral contact lenses. Contact Lens Anterior Eye. 2018;41(3):307–10.

97. Santos DL, Kwitko S, Marinho DR, Araújo BS, Locatelli CI, Rott MB. *Acanthamoeba* keratitis in Porto Alegre (southern Brazil): 28 cases and risk factors. Parasitol Res. 2018;117(3):747–50.

98. Possamai CO, Loss AC, Costa AO, Falqueto A, Furst C. *Acanthamoeba* of three morphological groups and distinct genotypes exhibit variable and weakly inter-related physiological properties. Parasitolol Res. 2018;117(5):1389–400.

99. Maschio JV, Virgínio VG, Ferreira HB, Rott MB. Comparative proteomic analysis of soluble and surface-enriched proteins from *Acanthamoeba castellanii* trophozoites. Mol Biochem Parasitol. 2018;225:47–53.

100. Buchele MLC, Wopereis DB, Casara F, Macedo JP, Rott MB, Monteiro FBF, et al. Contact lens-related polymicrobial keratitis : *Acanthamoeba* spp . genotype T4 and *Candida albicans*. Parasitol Res. 2018;117(11):3431–6.

101 Gonçalves DS, Ferreira MS, Liedke SC, Gomes KX, Oliveira GA, Leão PEL, et al. Extracellular vesicles and vesicle-free secretome of the protozoa *Acanthamoeba castellanii* under homeostasis and nutritional stress and their damaging potential to host cells. Virulence. 2018;9(1):2150–5608.

102. Machado ATP, Silva M, Iulek J. Structural studies of glyceraldehyde-3-phosphate dehydrogenase from *Naegleria gruberi*, the first one from phylum. Biochim Biophys Acta Proteins Proteom. 2018;1866:581–8.

103. Alves DSMM, Gonçalves GS, Moraes AS, Alves LM, Carmo Neto JR, Hecht MM, et al. The first *Acanthamoeba* keratitis case in the Midwest region of Brazil: diagnosis, genotyping of the parasite and disease outcome. Rev Soc Bras Med Trop. 2018;51(5):716–9.

104. Bellini NK, Santos TM, da Silva MTA, Thiemann OH. The therapeutic strategies against *Naegleria fowleri*. Exp Parasitol. 2018 Apr;187:1–11.

105. Fabres LF, Maschio VJ, Santos DL, Kwitko S, Marinho DR, Araújo BS, et al. Virulent T4 *Acanthamoeba* causing keratitis in a patient after swimming while wearing contact lenses in Southern Brazil. Acta Parasitol. 2018;63(2):428–32.

106 Zorzi GK, Schuh RS, Maschio VJ, Brazil NT, Rott MB, Teixeira HF. Box Behnken design of siRNA-loaded liposomes for the treatment of a murine model of ocular keratitis caused by *Acanthamoeba*. Colloids Surfaces B Biointerfaces. 2019;173:725–32.

107. Gonçalves DS, Ferreira MS, Guimarães AJ. Extracellular vesicles from the protozoa *Acanthamoeba castellanii*: Their role in pathogenesis, environmental adaptation and potential applications. Bioengineering. 2019;6(1):12.

108. Serrano-Solís V, Toscano Soares PE, de Farías ST. Genomic signatures among *Acanthamoeba polyphaga* entoorganisms unveil evidence of coevolution. J Mol Evol. 2019;87(1):7–15.

109. Medina G, Leýan P, Silva CV, Flores-Martin S, Manosalva C, Fernández H. Intra-amoebic localization of Arcobacter butzleri as an endocytobiont of *Acanthamoeba castellanii*. Arch Microbiol. 2019;201(10):1447–52.

110. Henker LC, Cruz RAS, Silva FS, Driemeier D, Sonne L, Uzal FA, et al. Meningoencephalitis due to *Naegleria fowleri* in cattle in southern Brazil. Rev Bras Parasitol Vet. 2019;28(3):514–7.

111. Corsaro D, Köhsler M, Venditti D, Rott MB, Walochnik J. Recovery of an *Acanthamoeba* strain with two group I introns in the nuclear 18S rRNA gene. Eur J Protistol. 2019;68:88–98.

112. Gonçalves DS, Ferreira MS, Gomes KX, La Noval CR, Liedke SC, Costa GCV, et al. Unravelling the interactions of the environmental host *Acanthamoeba castellanii* with fungi through the recognition by mannose - binding proteins. Cell Microbiol. 2019;21(10):e13066.

113. Faria LV, Carmo PHF, Costa MC, Peres NTA, Chagas IAR, Furst C, et al. *Acanthamoeba castellanii* as an alternative interaction model for the dermatophyte *Trichophyton rubrum*. Mycoses. 2020;63(12):1331–40.

114. Carvalho-Kelly LF, Pralon CF, Rocco-Machado N, Nascimento MT, Carvalho-de-Araújo AD, Meyer-Fernandes JR. *Acanthamoeba castellanii* phosphate transporter (AcPHS) is important to maintain inorganic phosphate influx and is related to trophozoite metabolic processes. J Bioenerg Biomembr. 2020;52(2):93–102.

115. Weber-Lima MM, Prado-Costa B, Becker-Finco A, Costa AO, Billilad P, Furst C, et al. *Acanthamoeba* spp . monoclonal antibody against a CPA2 transporter : a promising molecular tool for acanthamoebiasis diagnosis and encystment study. Parasitology. 2020;147(14):1678–88.

116. Cirelli C, Mesquita EIS, Chagas IAR, Furst C, Possamai CO, Abrahão JS, et al. Extracellular protease profile of *Acanthamoeba* after prolonged axenic culture and after interaction with MDCK cells. Parasitol Res. 2020;119(2):659–66.

117. Wopereis DB, Bazzo ML, De Macedo JP, Casara F, Golfeto L, Venancio E, et al. Free-living amoebae and its relationship with air quality in hospital environments: isolation and characterization of *Acanthamoeba* spp. from air-conditioning system. Parasitology. 2020;147(7):789–90.

118. Fonseca JDG, Gómez-Hernández C, Barbosa CG, Rezende-Oliveira K. Identification of T3 and T4 genotypes of *Acanthamoeba* sp. in dust samples isolated from air conditioning equipment of public hospital of Ituiutaba-MG. Curr Microbiol. 2020;77(5):890–5.

119. Fabres LF, da Costa Gonçalves F, Duarte EOS, Berté FK, da Conceição DKS lva, Ferreira LA, et al. *In vitro* amoebicidal activity of imidazolium salts against trophozoites. Acta Parasitol 2020;65(2):317–26.

120. Bellini NK, da Fonseca ALM, Reyes-Batlle M, Lorenzo-Morales J, Rocha O, Thiemann OH. Isolation of *Naegleria* spp. from a Brazilian water source. Pathogens. 2020;9(2):90.

121. Marujo FI, Hirai FE, Yu MCZ, Hofling-Lima AL, de Freitas D, Sato EH. Distribution of infectious keratitis in a tertiary hospital in Brazil. Arq Bras Oftalmol. 2013;76(6):370–3.

122. Moriyama AS, Hofling-Lima AL. Contact lens-associated microbial keratitis. Arq Bras Oftalmol. 2008;71(7):32–6.

123. Santos A, Silva LD, Sousa LB, de Freitas D, Oliveira LA. Results with the Boston Type I keratoprosthesis after *Acanthamoeba* keratitis. Am J Ophtalmol Case Rep. 2017;6:71–3.

124. Farias R, Pinho L, Santos R. Epidemiological profile of infectious keratitis. Rev Bras Oftalm. 2017;76(3):116–20.

125. Müller RT, Abedi F, Cruzat A, Witkin D, Baniasadi N, Cavalcanti BM, et al. Degeneration and regeneration of subbasal corneal nerves after infectious keratitis: a longitudinal in vivo confocal microscopy study. Ophthalmology. 2015;122(11):2200–9.

126. Frade MTS, Ferreira JS, Nascimento MJR, Aquino VVF, Macêdo IL, Carneiro RS, et al. Central nervous system disorders diagnosed in dogs. Pesq Vet Bras. 2018;38(5):935–48.

127. Boratto PVM, Oliveira GP, Machado TB, Andrade ACSP, Baudoin J-P, Klose T, et al. Yaravirus: A novel 80-nm virus infecting *Acanthamoeba castellanii*. Proc Natl Acad Sci. 2020;117(28):202001637.

128. Perim LV, Custódio NCC, Lima VCV, Igreja JASL, Alves DSMM, Storchilo HR, et al. Occurrence of parasites in salads in restaurants in Aparecida de Goiânia, Goiás, Brazil. J Trop Pathol Vol. 2020;49(3):207–14.

129. Leal DAG, Souza DSM, Caumo KS, Fongaro G, Panatieri LF, Durigan M, et al. Genotypic characterization and assessment of infectivity of human waterborne pathogens recovered from oysters and estuarine waters in Brazil. Water Res. 2018;137:273–80.

130. Silva LKDS, Rodrigues RAL, Andrade ACDSP, Hikida H, Andreani J, Levasseur A, et al. Isolation and genomic characterization of a new mimivirus of lineage B from a Brazilian river. Arch Virol. 2020;165(4):853–63.

131. Albuquerque P, Nicola AM, Magnabosco DAG, da Silveira Derengowski L, Crisóstomo LS, Xavier LCG, et al. A hidden battle in the dirt: soil amoebae interactions with *Paracoccidioides* spp. PLoS Negl Trop Dis. 2019;13(10):e0007742.

132. Rodrigues RAL, Abrahão JS, Drumond BP, Kroon EG. Giants among larges : how gigantism impacts giant virus entry into amoebae. Curr Opin Microbiol. 2016;31(31):88–93.

133. Gasparetto EL, Cabral RF, Hygino LC, Domingues RC. Diffusion imaging in brain infections. Neuroimaging Clin NA. 2011;21(1):89–113.

134. Abrahão JS, Dornas FP, Silva LCF, Almeida GM, Boratto PVM, Colson P, et al. *Acanthamoeba polyphaga* mimivirus and other giant viruses: an open field to outstanding discoveries. Virol J. 2014;11:1–12.

135. Hofstatter PG, Ribeiro GM, Porfírio-Sousa AL, Lahr DJG. The sexual ancestor of all eukaryotes: a defense of the "meiosis toolkit": a rigorous survey supports the obligate link between meiosis machinery and sexual recombination. BioEssays. 2020;42(9):1–10.

136. Rolland C, Andreani J, Louazani AC, Aherfi S, Francis R, Rodrigues R, et al. Discovery and further studies on Giant viruses at the IHU Mediterranee infection that modified the perception of the virosphere. Viruses. 2019;(11), 312.

137. Raoult D, Abrahão J. Editorial overview : the megaviromes. Curr Opin Microbiol. 2016;31:31–3.

138. Yin R, Dai T, Avci P, Jorge AES, Melo WCMA, Vecchio D, et al. Light based anti-infectives: ultraviolet C irradiation, photodynamic therapy, blue light, and beyond. Curr Opin Pharmacol. 2013;13(5):731–62.

139. Kollipara R, Peranteau AJ, Nawas ZY, Tong Y, Woc-colburn L, Yan AC, et al. Emerging infectious diseases with cutaneous manifestations: fungal, helminthic, protozoan and ectoparasitic infections. J Am Acad Dermatol. 2016;75(1):19–30.

140. Chimelli L. Co-infection of HIV and tropical infectious agents that affect the nervous system. Rev Neurol (Paris). 2012;168(3):270–82.

141. Sullivan KE, Bassiri H, Bousfiha AA, Costa-Carvalho BT, Freeman AF, Hagin D, et al. Emerging infections and pertinent infections related to travel for patients with primary immunodeficiencies. J Clin Immunol. 2017;37:650–92.

142. Chimelli L. A morphological approach to the diagnosis of protozoal infections of the central nervous system. Patholog Res Int. 2011;2011:29085.

143. Oliveira G, La Scola B, Abrahão J. Giant virus vs amoeba: fight for supremacy. Virol J. 2019;16(1):1–12.

144. Abrahão J, La Scola B. Editorial: large and giant DNA viruses. Front Microbiol. 2019;10(July):1–2.

145. Mougari S, Bekliz M, Abrahao J, Pinto F Di, Levasseur A, La Scola B. Guarani Virophage , a new sputnik-like isolate from a Brazilian lake. Front Microbiol. 2019;10(3).

146. Silva LCF, Araújo R, Rodrigues L, Oliveira GP, Dornas FP, La Scola B, et al. Microscopic analysis of the Tupanvirus cycle in *Vermamoeba vermiformis*. 2019;10(April):1–9.

147. Abrahão J, Silva L, Oliveira D, Almeida G. Lack of evidence of mimivirus replication in human PBMCs. Microbes Infect. 2018; May;20(5):281-283.

148. Andrade ACDSP, Rodrigues RAL, Oliveira GP, Andrade KR, Bonjardim CA, La Scola B, et al. Filling gaps about mimivirus entry, uncoating and morphogenesis. J Virol. 2017;22:e01355-17.

149. Hori JI, Pereira MSF, Roy CR, Nagai H, Zamboni DS, Celular B, et al. Identification and functional characterization of K(+) transporters encoded by *Legionella pneumophila* kup genes. Cell Microbiol. 2013;15(12):2006–19.

150. Medina G, Neves P, Flores-Martin S, Manosalva C, Andaur M, Otth C, et al. Transcriptional analysis of flagellar and putative virulence genes of *Arcobacter butzleri* as an endocytobiont of *Acanthamoeba castellanii*. Arch Microbiol. 2019;201(8):1075–83.

151. Dornas FP, Silva LCF, Almeida GM De, Campos RK, Boratto PVM, Franco-Luiz APM, et al. *Acanthamoeba* polyphaga mimivirus stability in environmental and clinical substrates: implications for virus detection and isolation. PLoS One. 2014;9(2):16–8.

152. Silva A, Nobre HJr, Sampaio L, Nascimento B, Silva C, de Andrade Neto JB, et al. Antifungal and antiprotozoal green amino acid-based rhamnolipids: mode of action , antibiofilm efficiency and selective activity against resistant *Candida* spp . strains and *Acanthamoeba castellanii*. Colloids Surfaces B Biointerfaces. 2020;193:111148.

153. Rodrigues RAL, Louazani AC, Picorelli A, Oliveira GP, Lobo FP, Colson P, et al. Analysis of a Marseillevirus transcriptome reveals temporal gene expression profile and host transcriptional shift. Front Microbiol. 2020;11(April):1–17.

154. Tavares PL, Ribeiro AC, Berte FK, Hellwig AH S, Pagani DM, Souza CCT, et al. The interaction between *Sporothrix schenckii* sensu stricto and *Sporothrix brasiliensis* with *Acanthamoeba castellanii*. Mycoses. 2020;63(3):302–7.

155. Pereira Andrade ACS, Boratto PVM, Rodrigues RAL, Bastos TM, Azevedo BL, Dornas FP, et al. New isolates of Pandoraviruses: contribution to the study of replication cycle steps. J Virol. 2019;93(5):1–12.

156. Mougari S, Abrahao J, Oliveira GP, Bou Khalil JY, La Scola B. Role of the R349 gene and its repeats in the MIMIVIRE defense system. Front Microbiol. 2019;10(May):1–10.

157. Borges IA, Assis FL, Silva LKDS, Abrahão J. Rio Negro virophage: sequencing of the near complete genome and transmission electron. Brazilian J Microbiol. 2018;49(Suppl 1):260–1.

158. Boratto PVM, Dornas FP, Silva LCF, Rodrigues RAL, Oliveira GP, Cortines JR, et al. Analyses of the Kroon virus major capsid gene and its transcript highlight a distinct pattern of gene evolution and splicing among Mimiviruses. J Virol. 2018;92(2):1–11.

159. Oliveira GP, Lima MT, Arantes TS, Assis FL, Rodrigues RAL, da Fonseca FG, et al. The investigation of promoter sequences of Marseilleviruses highlights a remarkable abundance of the AAATATTT motif in intergenic regions. J Virol. 2017;91(21):1–10.

160. Rizzo J, Albuquerque PC, Wolf JM, Nascimento R, Pereira MD, Nosanchuk JD, et al. Analysis of multiple components involved in the interaction between *Cryptococcus neoformans* and *Acanthamoeba castellanii*. Fungal Biol. 2017;121(6–7):602–14.

161. Schrad JR, Young EJ, Abrahão JS, Cortines JR, Parent KN. Microscopic characterization of the Brazilian giant samba virus. Viruses. 2017;9(30):1–16.

162. Arantes TS, Rodrigues RAL, dos Santos Silva LK, Oliveira GP, de Souza HL, Khalil JYB, et al. The large Marseillevirus explores different entry pathways by forming giant infectious vesicles. J Virol. 2016;90(11):5246–55.

163. Decarli MC, Carvalho MT, Corrêa TQ, Bagnato VS, Souza CWO de. Different photoresponses of microorganisms: from bioinhibition to biostimulation. Curr Microbiol. 2016;72(4):473–81.

164. Dornas FP, Assis FL, Aherfi S, Arantes T, Abrahão JS, Colson P, et al. A Brazilian Marseillevirus is the founding member of a lineage in family Marseilleviridae. Viruses. 2016;8(76):1–16.

165. Rodrigues RAL, Silva LKS, Dornas FP, Oliveira DB, Magalhães TFF, Santos DA, et al. *Mimivirus fibrils* are important for viral attachment to the microbial world by a diverse glycoside interaction repertoire. J Virol. 2015;89(23):11812–9.

166. Boratto PVM, Arantes TS, Silva LCF, Assis FL, Kroon EG, La Scola B, et al. Niemeyer virus: a new mimivirus group A isolate harboring a set of duplicated aminoacyl-tRNA synthetase genes. Front Microbiol. 2015;6(NOV):1–11.

167. Campos RK, Boratto P V, Assis FL, Aguiar ERGR, Silva LCF, Albarnaz JD, et al. Samba virus: A novel mimivirus from a giant rain forest, the Brazilian Amazon. Virol J. 2014;11(1):1–11.

168. Boratto PVM, Dornas FP, Andrade KR, Rodrigues R, Peixoto F, Silva LCF, et al. Amoebas as mimivirus bunkers: increased resistance to UV light, heat and chemical biocides when viruses are carried by amoeba hosts. Arch Virol. 2014;159(5):1039–43.

169. Silva LCF, Almeida GMF, Oliveira DB, Dornas FP, Campos RK, La Scola B, et al. A resourceful giant: APMV is able to interfere with the human type I interferon system. Microbes Infect. 2014;16(3):187–95.

170. Lopes FC, Tichota DM, Sauter IP, Meira SMM, Segalin J, Rott MB, et al. Active metabolites produced by *Penicillium chrysogenum* IFL1 growing on agro-industrial residues. Ann Microbiol. 2013;63:771–8.

171. Derengowski LS, Paes HC, Albuquerque P, Tavares AHFP, Fernandes L, Silva-Pereira I, et al. The transcriptional response of *Cryptococcus neoformans* to ingestion by *Acanthamoeba castellanii* and macrophages provides insights into. Eukaryot Cell. 2013;12(5):761–74.

172. Campos RK, Andrade KR, Ferreira PCP, Bonjardim CA, Scola B La, Kroon EG, et al. Virucidal activity of chemical biocides against mimivirus , a putative pneumonia agent. J Clin Virol. 2012;55(4):323–8.

173. Souza F, Rodrigues R, Reis E, Lima M, La Scola B, Abrahão J. In-depth analysis of the replication cycle of *Orpheovirus*. Virol J. 2019;16(1):1–11.

174. Borges I, Rodrigues RAL, Dornas FP, Almeida G, Aquino I, Bonjardim CA, et al. Trapping the enemy: *Vermamoeba vermiformis* circumvents *Faustovirus mariensis* dissemination by enclosing viral progeny inside cysts . J Virol. 2019;93(14):1–19.

175. Augusto G, Souza P, Queiroz VF, Lima MT, Vinicius E, Reis DS, et al. Virus goes viral: an educational kit for virology classes. Virol J. 2020;17(1):1–8.

176. Abrahão J, Silva L, Silva LS, Khalil JYB, Rodrigues R, Arantes T, et al. Tailed giant Tupanvirus possesses the most complete translational apparatus of the known virosphere. Nat Commun . 2018;9(1).

177. Dornas FP, Khalil JYB, Pagnier I, Raoult D, Abrahão J, La Scola B. Isolation of new Brazilian giant viruses from environmental samples using a panel of protozoa. Front Microbiol. 2015;6(October):1–9.

178. Abrahão JS, Boratto P, Dornas FP, Silva LC, Campos RK, Almeida GMF, et al. Growing a giant: evaluation of the virological parameters for mimivirus production. J Virol Methods. 2014;207:6–11.
